# Heart Rate Monitors for the Estimation of Physical Activity in Patients With Cardiovascular Disease: Systematic Review

**DOI:** 10.2196/79995

**Published:** 2026-06-17

**Authors:** Paulien Vermunicht, Christophe Buyck, Sebastiaan Naessens, Wendy Hens, Emeline Van Craenenbroeck, Kris Laukens, Lien Desteghe, Hein Heidbuchel

**Affiliations:** 1 Research Group Cardiovascular Diseases, University of Antwerp, Drie Eikenstraat 655, Antwerp, 2650, Belgium, 3238212195; 2 Department of Cardiology, Antwerp University Hospital, Drie Eikenstraat 655, Antwerp, 2650, Belgium; 3 Research Group Movant, University of Antwerp, Antwerp, Belgium; 4 Department of Computer Science, University of Antwerp, Antwerp, Belgium; 5 Biomedical Informatics Research Network Antwerp (Biomina), University of Antwerp, Antwerp, Belgium; 6 Faculty of Medicine and Life Sciences, Hasselt University, Hasselt, Belgium; 7 Center for Research and Innovation in Care, University of Antwerp, Antwerp, Belgium

**Keywords:** exercise, heart rate, wearable electronic devices, fitness trackers, cardiac rehabilitation

## Abstract

**Background:**

Heart rate (HR) monitoring by wearable devices offers a physiological, personalized, and continuous method for assessing physical activity (PA) duration and intensity. However, methods to translate HR data into meaningful PA metrics are diverse and nonstandardized.

**Objective:**

This study aims to provide an overview of how HR data are used to quantify PA behavior and estimate physiological outcomes in adult patients with cardiovascular disease (CVD).

**Methods:**

A systematic search was performed in PubMed, Web of Science, and CENTRAL for studies published between 2014 and 2024. Eligible studies included adults with CVD or related risk factors wearing HR monitors to estimate PA. Data were synthesized narratively. The methodological quality of the included studies was evaluated using the Crowe Critical Appraisal Tool (CCAT; Michael Crowe).

**Results:**

Twenty studies were included, spanning four HR-based PA estimation methods: (1) HR zone analysis (n=14), which assessed time spent in moderate-to-vigorous zones to evaluate guideline or training adherence; (2) physiological modeling (n=4), estimating outcomes such as energy expenditure (physical activity level) or cardiorespiratory fitness (maximal oxygen uptake); (3) change detection (n=1), using time-series and machine learning algorithms to quantify shifts in PA behavior; and (4) a derived personalized scoring system (n=1). While each approach demonstrated clinical promise of using HR data, external validation, and methodological transparency is often lacking.

**Conclusions:**

HR-based PA estimation holds the promise of physiologically meaningful, personalized PA monitoring in CVD care. Modeling approaches and personalized scoring systems linking PA behavior to cardiovascular outcomes may provide highly needed clinical tools for PA management in patients. Research should prioritize algorithm transparency, clinical validation, and standardization.

## Introduction

Engaging in sufficient physical activity (PA) is integral to the management of cardiovascular diseases (CVDs). The latest guidelines recommend ≥150 min/week of moderate-intensity PA, ≥75 min/week of vigorous-intensity PA, or an equivalent combination of both, typically expressed as the weekly accumulation of moderate-to-vigorous physical activity (MVPA), which can often be quantified using physiological indicators such as heart rate intensity zones [1]. PA is associated with reduction in cardiovascular and all-cause mortality, improvement in functional capacity and quality of life, and better control of key risk factors such as hypertension, dyslipidemia, and diabetes [1-4]. Despite the proven benefits of exercise-based cardiac rehabilitation (CR) in patients with CVD, with reductions of future cardiac events [5-7], its long-term effectiveness is often limited by low adherence to recommended PA levels and inadequate monitoring of whether patients adhere to recommended PA levels, particularly when transitioning from supervised clinical settings (ie, phase 2 CR) to independent activities at home in phase 3 [8,9]. Nonadherence leads to a decline in cardiovascular function and undermines the potential long-term benefits of CR programs [10,11].

Continuous, accurate, and noninvasive monitoring of PA during patients’ everyday activities could provide a solution to this challenge [12]. However, traditional PA monitoring methods such as self-reported questionnaires or activity logs are often subjective and prone to recall bias, and may not accurately reflect PA intensity [13,14]. Even objective methods like accelerometry primarily capture movement quantity (eg, steps and counts) and may approximate intensity (eg, via step cadence), but often do not adequately reflect physiological effort, making them less suited to fully assess PA intensity in clinical populations. In contrast, (cardiopulmonary) exercise testing or 6-minute walk tests offer objective insights but are limited to single time points and are impractical for frequent or real-world use [15,16].

Heart rate (HR) based PA estimation holds the promise of physiologically meaningful, personalized PA monitoring in CVD care. HR reflects the body’s physiological response to activity intensity and is directly related to oxygen consumption and cardiac output [17]. Unlike movement-based metrics such as step count or distance walked, HR provides a direct measure of the body’s metabolic demand and reaction to activity intensity, regardless of the type of exercise [18]. In line with clinical guidelines, PA intensity is often prescribed using target HR zones, especially in CR contexts [1,19]. This makes HR a more precise and individualized indicator of exertion and cardiorespiratory demand, and thus a uniquely valuable parameter for quantifying PA [19-21]. Advances in wearable technology have made near-continuous HR monitors, such as photoplethysmography-based wrist-worn devices and chest straps, accessible tools for continuous, noninvasive tracking of HR in everyday environments, provided they are properly validated.

However, translating HR data into meaningful, personalized metrics that guide patients and physicians toward adequate PA and optimal health outcomes remains a challenge. Although wearable devices can provide continuous HR measurements, there is a lack of standardized, transparent methods for interpreting this data in ways that are clinically actionable and tailored to patients’ individual needs. While commercial devices such as those from Fitbit (Fitbit Inc) or Garmin (Garmin Ltd) often provide proprietary activity scores or readiness indicators, these metrics are typically based on “black box” algorithms that often rely more on accelerometer data than on HR data, and are rarely validated in clinical populations such as patients with CVD. Consequently, their clinical utility and safety in guiding PA remain uncertain.

The primary aim of this review is to explore how wearable HR monitors, including wrist-worn photoplethysmography sensors and electrocardiogram (ECG)-based chest straps across consumer-grade and research or medically validated devices, have been used to estimate and quantify PA with methodologies relevant to patients with CVD. Specifically, this review seeks to classify and describe the methodological approaches used to translate HR data into PA metrics and physiological estimates, including adherence rates and personalized scoring systems, across CR and broader CVD populations. In contrast to prior reviews that addressed wearable technologies in (tele) CR more broadly [22], this review focuses specifically on the methodological frameworks used to derive clinically meaningful PA indicators from HR data. By providing an overview of these methodologies, the review aims to offer insights into the availability and potential gaps in methods for HR-based PA monitoring in this population.

## Methods

### Overview

The PRISMA (Preferred Reporting Items for Systematic Reviews and Meta-Analyses) 2020 statement was followed in conducting this review (Checklist 1) [23]. This systematic review was not prospectively registered. A protocol was developed by the authors to guide the review process, but it was not published or made publicly available.

### Search Strategy

A comprehensive search was conducted in PubMed, Web of Science, and the Cochrane Central Register of Controlled Trials (CENTRAL) on April 3, 2024 to identify relevant studies. The search strategy was developed based on the PICO (population, intervention, comparison, outcome) framework [24] and combined terms related to “CVD,” “HR,” “wearable HR monitors,” and “PA” using Boolean operators. Search syntaxes were adapted for each database to account for differences in indexing and Boolean operator functionalities. Full search strings for each database are detailed in Table S1 in Multimedia Appendix 1. In addition to database searches, the reference lists of included articles were screened to identify additional relevant studies (backward citation search).

### Eligibility Criteria

Studies were eligible for this review if they met the following inclusion criteria*.*

#### Population

Studies had to focus on adults aged 18 years or older with CVD. CVD was defined broadly and included individuals with coronary artery disease (CAD), myocardial infarction, acute coronary syndrome (ACS), heart failure with preserved or reduced ejection fraction (HFpEF/HFrEF), stroke, congenital heart disease, valvular diseases, and postrevascularization conditions (eg, percutaneous coronary intervention [PCI], coronary artery bypass grafting [CABG]). Studies conducted in patients with cardiovascular risk factors (eg, hypertension, diabetes, and metabolic syndrome) and in healthy populations were also considered if the authors explicitly stated that the HR-based PA estimation method described in the study was specifically intended or useful for application in cardiac populations.

#### HR-Based PA Estimation

Studies had to use wearable HR monitors (photoplethysmography-based wrist-worn devices or ECG-based chest straps) to estimate or quantify PA.

#### Study Types and Publication Criteria

Eligible studies included original research articles and study protocols. Protocols were included if they provided detailed methodological descriptions on how HR monitors or HR-based PA metrics would be used to assess PA, even if no results were reported. Including protocols allowed for the capture of methodological advancements relevant to the primary aim of the review. To ensure technological relevance, studies had to be published within the last 10 years (ie, between January 2014 and April 2024). Furthermore, only studies published in English with full-text availability were eligible.

#### Exclusion Criteria

Studies were excluded if HR monitors were solely used for exercise prescription or guidance, such as setting HR zones for training sessions, without quantifying or evaluating PA based on HR data. Studies were also excluded if they did not focus on a cardiac population or did not describe an HR-based PA estimation method applicable to cardiovascular health. Additionally, review articles, editorials, and conference abstracts were not eligible. Studies were also excluded if they were not published in English, were published more than 10 years ago, or lacked full-text availability.

### Screening and Data Management

Duplicate records were identified and removed prior to screening, using a systematic deduplication method in EndNote, as described by Bramer et al [25] The screening process was conducted in multiple stages using Rayyan software (Rayyan Systems Inc.). Initially, 2 reviewers (PV and CB) independently screened article titles to exclude irrelevant studies. This was followed by a screening of abstracts. Articles deemed relevant based on abstract screening proceeded to full-text review, during which the reviewers assessed the complete articles to confirm their eligibility according to the predefined criteria. Studies where eligibility could not be determined from the abstract were included for full-text review. If discrepancies arose at any stage, they were resolved through discussion or, if necessary, a third reviewer (LD) was consulted to resolve them.

### Data Extraction and Outcomes of Interest

A structured data extraction form was developed in Microsoft Excel and pilot-tested on 2 randomly selected studies to ensure consistent, clear, and comprehensive data collection. Two independent reviewers (PV and CB) extracted data from the included studies and any discrepancies were resolved through consensus discussions between the 2 reviewers (PV and CB); and if necessary, a third reviewer (LD) was consulted.

The primary outcome of interest in this review was the methodological approach used to translate wearable HR data into PA-related metrics or physiological estimates. Specifically, we extracted and classified how HR data were operationalized (eg, threshold-based HR zone analysis, regression-based energy expenditure models, cardiorespiratory fitness estimation, change detection algorithms, or personalized activity scores).

In addition, extracted data included study characteristics (eg, title, authors, publication year, country, funding, and design), participant demographics, HR monitor specifications, and details on the intervention or observation period (eg, training program and duration). We also extracted study-specific reported outcomes to provide contextual interpretation of each methodological approach. The reported outcome parameters varied across studies and included measurement validity indicators (eg, comparison with CPET or ECG, mean absolute [percentage] error, intraclass correlation coefficient, coefficient of determination), adherence rates, physiological outcomes (eg, maximal oxygen uptake [VO_₂_max]), feasibility metrics, and safety events. Given the heterogeneity in devices, populations, and study designs, these outcomes were synthesized descriptively and were not pooled quantitatively.

### Methodological Quality Assessment

The methodological quality of the included studies was evaluated using the Crowe Critical Appraisal Tool (CCAT; Michael Crowe) [26]. This tool was selected due to its flexibility and applicability across diverse study designs, making it particularly suitable for this review, which included randomized controlled trials, observational studies, feasibility studies, methodological studies, and protocol descriptions.

The CCAT assessed studies across 8 domains, including preliminaries, introduction, design, sampling, data collection, ethics, results, and discussion. Each domain is scored on a scale from 0 to 5, where 5 indicates that the criterion is fully met and 0 indicates that it is not addressed. Intermediate scores reflect partial fulfillment of the domain criteria according to the CCAT user guide [27]. Domain scores were assigned using the guidance provided in the CCAT user guide, where item descriptors support reviewer judgment when determining the final domain score [27]. The maximum total score is 40.

For protocol descriptions, only applicable domains of the CCAT were scored. Domains that were not applicable due to the absence of empirical results (eg, results and discussion) were marked as “not applicable”. The total score for protocols was recalculated as a percentage of the maximum applicable score (eg, if 6 domains were scored, the total score was calculated out of 30 instead of 40). This approach ensured that protocol descriptions were fairly assessed for their methodological relevance without being penalized for the absence of empirical findings.

Two independent reviewers (PV and CB) assessed methodological quality and resolved discrepancies through consensus discussion. CCAT scores did not influence study inclusion decisions and were used to contextualize interpretation rather than to weight or exclude findings within the narrative synthesis.

Although the CCAT is primarily designed as a methodological appraisal tool, several domains address aspects closely related to potential sources of bias (eg, study design, sampling strategy, data collection procedures, and discussion of bias and confounding). Therefore, CCAT scores provide an indirect indication of methodological robustness and susceptibility to bias.

### Data Synthesis

Given the heterogeneity of study designs and outcomes, a narrative synthesis was conducted. The primary aim of the synthesis was to identify and classify methodological approaches used to translate HR data into PA-related metrics.

During data extraction, the reported HR-based PA estimation methods from each study were examined and compared. Through an inductive process, recurring methodological approaches were identified and grouped into conceptual categories based on their underlying estimation strategy. This resulted in 4 distinct categories, explained in section “Heart Rate–Based Physical Activity Estimation Methods” of the Results. The categorization was discussed between the 2 reviewers (PV and CB) and finalized through consensus.

After categorization, studies within each methodological group were compared descriptively with respect to contextual characteristics such as HR monitor type, study population, and reported outcomes. Similarities and differences between studies were identified through iterative comparison across these dimensions. Methodological gaps were defined as areas where approaches lacked transparency, validation in cardiovascular populations, or consistent methodological reporting.

## Results

### Study Selection Process

**Figure 1.**
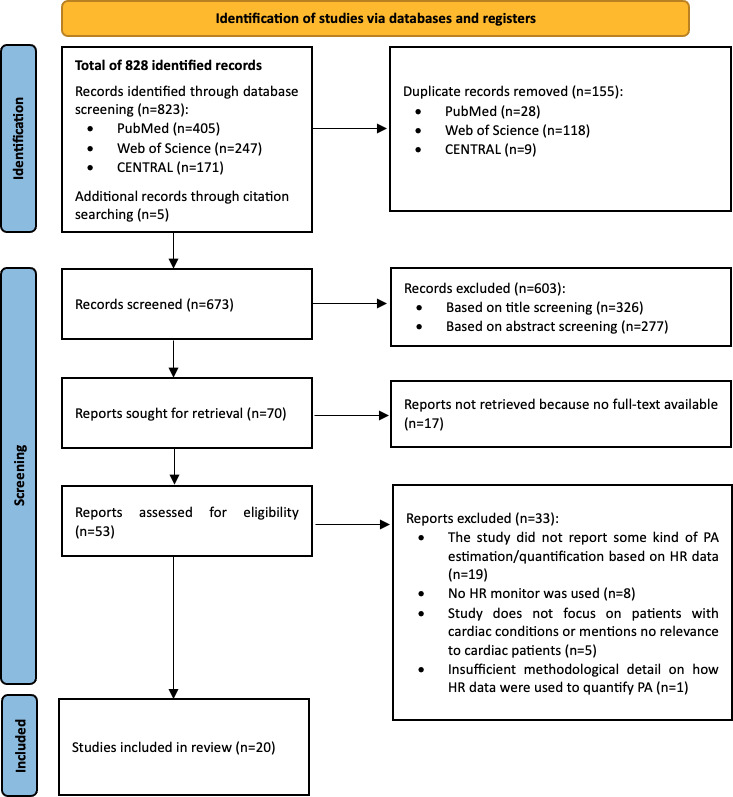
PRISMA (Preferred Reporting Items for Systematic Reviews and Meta-Analyses) flow diagram of the selection process. HR: heart rate; PA: physical activity.

The study selection process is summarized in a PRISMA 2020 flow diagram (Figure 1). The search for studies quantifying PA using HR monitors in patients with CVD yielded 828 results. After removal of duplicates, 673 publications were screened first on title and then on abstract. A total of 53 articles were screened on full text, 20 of which met the inclusion criteria.

### General Study Characteristics

Of the 20 included studies, 15 [28-42] were research articles and 5 [43-47] were protocol papers (Table 1, left side). The study designs included 8 [28,29,38,43-47] randomized controlled trials (RCTs), 5 [30-33,42] nonrandomized interventional studies (4 feasibility studies and 1 pre-post study), and 7 [34-37,39-41] observational studies that monitored PA patterns using HR monitors without aiming to induce behavioral change. Studies were conducted across diverse geographical locations, primarily in Europe and the United Kingdom (n=12) [29,31,32,35,36,38-40,43,45-47], followed by North America (n=5) [28,34,37,41,44], Asia (n=2) [30,33], and Australia (n=1) [42]. Sample sizes varied widely, with feasibility studies including the fewest participants (range: 5‐31), whereas other study designs included up to 300 participants.

**Table 1.** General study characteristics and population descriptions.

		General study characteristics					Study populations characteristics				
Study ID	First author (year)	Article type	Study design	Center type	Study country	Sample size (n)	Population	Gender distribution, n (%)	Age (years) ^a^	BMI (kg/m²),^a^	Beta-Blocker use, n (%)
1	Alonso et al (2021) [28] *HEART Camp*	Research article	RCT^b^	Multicenter	United States	204	Patients with heart failure (HFrEF^c^ and HFpEF^d^)	Female: 91 (45)^e^ Male: 113 (55)^e^	HFpEF (intervention): 63.3 (9.4)^e^ HFpEF (control): 65.6 (9.3)^e^ HFrEF (intervention): 58.6 (13.3)^e^ HFrEF (control): 58.6 (10.1)^e^	HFpEF (intervention): 35.6 (7.2)^e^ HFpEF (control): 36.4 (8.0)^e^ HFrEF (intervention): 34.7 (9.0)^e^ HFrEF (control): 33.9 (7.6)^e^	199 (98)
2	Crozier et al (2023) [43] *MOTIVATE-CR+*	Protocol paper	RCT (feasibility)	Multicenter	United Kingdom	72	Patients post myocardial infarction	—^f^	—	—	—
3	Mansfield et al (2017) [44] *PROPEL*	Protocol paper	RCT	Multicenter	Canada	192	Patients who have experienced stroke	—	—	—	—
4	Batalik et al (2020) [29]	Research article	RCT	Single-center	Czech Republic	51	Patients undergoing CR^g^ (ACS^h^, LVEF^i^>45%, and post heart revascularization)	Female: 9 (18)^e^ Male: 42 (82)^e^	Intervention: 56.5 (6.9)^e^ Control: 57.7 (7.6)^e^	Intervention: 28.0 (3.5)^e^ Control: 28.3 (4.3)^e^	46 (90)
5	Chung et al (2019) [30]	Research article	Feasibility (interventional, non-RCT)	Single-center	South Korea	16	Healthy adults^j^	Female: 11 (69)^e^ Male: 5 (31)^e^	26.3 (4.3)^e^	Not disclosed	Not disclosed
6	Dobrican and Zampunieris (2016) [31]	Research article	Feasibility (interventional, non-RCT)	Single-center	Luxembourg	5	Healthy adults^j^	Female: 0 (0)^e^ Male: 5 (100)^e^	21.0‐48.0^k^	Not disclosed	0 (0)
7	Dosbaba et al (2020) [45]	Protocol paper	RCT	Single-center	Czech Republic	76	Patients undergoing CR (ACS, LVEF>40%, post heart revascularization)	—	—	—	—
8	Quero et al (2017) [32]	Research article	Feasibility (interventional, non-RCT)	Single-center	Spain	Not explicitly disclosed^c^	Patients undergoing CR (CAD^l^, low-risk)	Not disclosed^m^	—	—	—
9	Suchy et al (2014) [46] *OptimEx-CLIN*	Protocol paper	RCT	Multi-center	Germany, Norway, Austria, Belgium	180	Patients with heart failure (HFpEF)	—	—	—	—
10	Xia et al (2023) [33]	Research article	Feasibility (interventional, non-RCT)	Single-center	China	31	Patients undergoing CR (CAD, ACS, and/or post heart revascularization)	Female:1 (3)^e^ Male: 30 (97)^e^	56.2 (13.4)^e^	26.2 (2.9)^e^	20 (65)
11	Koffman et al (2023) [34]	Research article	Observational (prospective)	Single-center	United States	70	Patients who have experienced stroke	Female: 32 (46)^e^ Male: 38 (54)^e^	61.0 (13.0)^e^	Not disclosed	42 (60)
12	Schubert et al (2020) [35]	Research article	Observational (prospective)	Multi-center	United Kingdom, Germany	107 datasets from 84 patients	Valve disease patients (mitral or aortic)	Female: 33 (39)^e^ Male: 51 (61)^e^	66.0 (IQR 56.0‐75.0)^n^	26.8 (IQR 24.4‐29.5)^n^	58 (54)
13	Sandberg et al (2016) [36]	Research article	Observational (prospective)	Single-center	Sweden	122	Adults with congenital heart disease	Female: 48 (39)^e^ Male: 74 (61)^e^	Simple lesions: 30.0 (IQR 20.7)^n^ Complex lesions: 133.7 (IQR 29.4)^n^ Controls: 31.4 (IQR 24.1)^n^	Simple lesions: 25.1 (4.3)^e^ Complex lesions: 23.5 (4.0)^e^ Controls: 25.8 (5.3)^e^	18 (15)
14	Weeks et al (2018) [37]	Research article	Observational (prospective)	Single-center	USA	15	Patients undergoing inpatient rehabilitation (various conditions, including cardiac disorders and stroke)	Female: 7 (47)^e^ Male: 8 (53)^e^	44.0‐80.0^k^	Not disclosed	4 (27)
15	Brouwers et al (2017) [47] *SmartCare-CAD*	Protocol paper	RCT	Single-center	Netherlands	300	Patients undergoing CR (CAD, ACS, and/or post heart revascularization)	—	—	—	—
16	Kraal et al (2017) [38] *FIT@Home*	Research article	RCT	Single-center	Netherlands	90	Patients undergoing CR (ACS, and/or post heart revascularization)	Female: 10 (11)^e^ Male: 80 (89)^e^	59.2 (8.5)^e^	Intervention: 28.2 (3.9)^e^ Control: 27.8 (4.8)^e^	82 (91)
17	Kraal et al (2016) [39]	Research article	Observational (prospective)	Single-center	Netherlands	16	Patients undergoing CR (ACS, and/or post heart revascularization)	Female: 0 (0)^e^ Male: 16 (100)^e^	55.8 (7.3)^e^	Development: 28.6 (3.5)^e^ Validation: 30.7 (3.1)^e^	14 (88)
18	Rissanen et al (2022) [40] *HealthBeat*	Research article	Observational (prospective)	Single-center	Finland	74	Adults with cardiovascular risk factors	Female: 56 (76)^e^ Male: 18 (24)^e^	55.8 (IQR 35.0‐64.0)^n^	28.7 (IQR 21.9‐39.9)^n^	0 (0)
19	Sprint et al (2017) [41]	Research article	Observational (prospective)	Single-center	United States	24	Patients undergoing inpatient rehabilitation (various conditions, including cardiac disorders and stroke)	Female: 12 (50)^e^ Male: 12 (50)^e^	Rehab group: 61.3 (12.7)^e^ Control group: 21.2 (2.2)^e^	Not disclosed	4 (17)
20	Hannan et al (2021) [42]	Research article	Mixed methods pre-post study (interventional, non-RCT)	Single-center	Australia	18	Patients undergoing CR in maintenance phase (ACS, post heart revascularization and/or valvular surgery)	Female: 3 (17)^e^ Male: 15 (83)^e^	56.0 (15.5)^e^	26.5 (4.4)^e^	3 (17)

a For each study, age/BMI was reported as originally provided by the authors. If age/BMI was available for the total study population, this value was reported. If only group-specific data were available, separate values are shown.

b RCT: randomized controlled trial.

c HFrEF: heart failure with a reduced ejection fraction.

d HFpEF: heart failure with a preserved ejection fraction.

e Mean (SD).

f Not applicable; applies to protocol papers where no results were reported.

g CR: cardiac rehabilitation.

h ACS: acute coronary syndrome (ie, myocardial infarction or unstable angina).

i LVEF: left ventricular ejection fraction.

j The study was included because of its focus on developing a system specifically for patients undergoing CR, despite the feasibility here being tested in healthy individuals.

k Range

l CAD: coronary artery disease.

m Case-based evaluation with a limited/nonspecified number of scenarios.

n Median (IQR)

### Population Characteristics

An overview of the study populations is shown in Table 1 (right side). In terms of health characteristics, 8 studies [29,32,33,38,39,42,45,47] focused on patients eligible for CR following ACS, CAD, or revascularization procedures (eg, PCI or CABG). Other study populations included patients with heart failure (n=2) [28,46], post-stroke patients who have experienced a stroke (n=2) [34,44], individuals with cardiovascular risk factors (n=1) [40], congenital heart disease (n=1) [36], valve disease (n=1) [35], and patients postmyocardial infarction (n=1) [43]. Two studies [37,41] investigated inpatient rehabilitation patients with mixed conditions including cardiac disorders, while 2 feasibility studies [30,31] were conducted in healthy adults, assessing HR-based PA methods intended for populations with heart diseases.

CR studies had a predominantly male representation (82%‐100% [29,33,38,39,42]), whereas populations with heart failure (55% male [28]) and populations who have experienced strokes (54% male [34]) were more balanced. Age ranged from young healthy adults (mean 26.3, SD 4.3 years [30]) to older patients with heart failure (up to mean 65.6, SD 9.3 years [28]). Patients undergoing CR typically ranged between 55.8 (SD 7.3) years [39] and 59.2 (SD 8.5) years [38]. BMI values were generally between 26 and 31 kg/m² for CR populations [29,33,38,39,42], with the highest BMI observed in patients with heart failure (33.9‐36.4 kg/m² [28]). Beta-blocker use was the highest among patients with heart failure (98% [28]) and ranged between 17% and 91% in CR populations [29,33,38,39,42].

### HR Monitors

A total of 12 out of 20 studies [29-32,34,35,37,41-44,47] used a wrist-worn HR monitor (60%), 7 [33,36,38-40,45,46] used a chest-strap (35%), and 1 [28] did not specify the type (5%) (Table 2). Polar devices were the most commonly used (6 studies) [28,29,32,43,45,46], followed by Fitbit (4 studies) [34,37,41,44] and Garmin (2 studies) [38,39]. Other brands included Samsung, Philips, Firstbeat Technologies, and custom-designed wearables.

**Table 2.** Specifications of used heart rate monitors and heart rate-based physical activity estimation methods.

		HR monitor		HR^a^-based PA^b^ estimation		Other PA estimation present?	
Study ID	First author (year)	Type	Brand/ Specification	Category (subcategory)	Details	Accelerometer-based	Questionnaire-based
1	Alonso et al. (2021) [28], HEART Camp	Unspecified	Polar (only the brand mentioned, not the type)	HR zone analysis (PA adherence)	PA adherence is defined as: recording at least 120 minutes/week (ie, 80% of the recommended 150 minutes/week) of moderate intensity exercise (ie, 40%‐80% of HRR^c^) HRR (HRmax – HRrest) determination: CPET^d^-based	No	Yes
2	Crozier et al. (2023) [43], MOTIVATE-CR+	Wrist-worn^e^	Polar Ignite 2^e^	HR zone analysis (PA adherence)	PA adherence is defined as: recording at least 150 minutes/week of moderate intensity exercise (ie, 40%‐70% of HRR) HRR (HRmax – HRrest) determination: not disclosed	Yes	Yes
3	Mansfield et al. (2017) [44], PROPEL	Wrist-worn	Fitbit Charge	HR zone analysis (PA adherence)	PA adherence is defined as: 1) recording at least 150 minutes/week of moderate intensity exercise (ie, 55%‐80% of HRmax) and/or (2) recording at least 150 “Fitbit active minutes”/week HRmax determination: with age-dependent formula (specific formula not mentioned)	No	Yes
4	Batalik et al. (2020) [29]	Wrist-worn	Polar M430	HR zone analysis (training adherence)	Adherence to a structured training program with predefined frequency, intensity, and duration (eg, 3 × 60-minutes/week at 70%‐80% of HRR): Intensity assessment: time spent in prescribed HR zone and average training intensity (HRR) Quantitative assessment: number of completed sessions HRR (HRmax – HRrest) determination: CPET-based	No	No
5	Chung et al. (2019) [30]	Wrist-worn	Custom-designed wearable device	HR zone analysis (training adherence)	Adherence to a structured training program with predefined intensity (ie, 50%‐70% of HRmax): Intensity assessment: percentage of time within prescribed HR zone during exercise (referred to as ‘target heart rate retention ratio’) Quantitative assessment: number of completed sessions HRmax determination: CPET-based	No	No
6	Dobrican and Zampunieris (2016) [31]	Wrist-worn	Samsung Gear S2	HR zone analysis (training adherence)	Adherence to a structured training program with predefined intensity (ie, 65%‐90% HRmax): Intensity assessment: time spent in prescribed HR zones Quantitative assessment: number of completed sessions HRmax determination: with age-dependent formula (220 – age)	No	No
7	Dosbaba et al. (2020) [45]	Chest-strap	Polar H10	HR zone analysis (training adherence)	Adherence to a structured training program with predefined frequency, intensity, and duration (eg, 3×33-minutes/week at 85%‐95% of HRmax during intervals): Intensity assessment: time spent in prescribed HR zones Quantitative assessment: completion of ≥70% of prescribed sessions (regardless of HR) HRmax determination: CPET-based	No	No
8	Quero et al. (2017) [32]	Wrist-worn	Polar M600	HR zone analysis (training adherence)	Adherence to a structured training program with predefined intensity and duration (ie, 15‐40 min, target HR zones based on HRmax, HRrest, ventilatory thresholds): Intensity assessment: time spent in prescribed HR zones (classified via fuzzy logic algorithm) Quantitative assessment: number of completed sessions Target HR zone determination: CPET-based	No	No
9	Suchy et al. (2014) [46], OptimEx-CLIN	Chest-strap	Polar H7	HR zone analysis (training adherence)	Adherence to a structured training program with predefined frequency, intensity, and duration (eg, 3 × 38-minutes/week at 90%‐95% of HRmax during intervals): Intensity assessment: time spent in prescribed HR zones Quantitative assessment: completion of ≥70% of prescribed sessions (regardless of HR) HRmax determination: CPET-based	Yes	No
10	Xia et al. (2023) [33]	Chest-strap	Recovery Plus Inc	HR zone analysis (training adherence)	Adherence to a structured training program with predefined frequency, intensity, and duration (ie, 3‐5 × 30-60-minutes/week, target HR=anaerobic threshold±5‐10 bpm): Intensity assessment: time spent in prescribed HR zones Quantitative assessment: adherence defined as ≥10 minutes/day in target HR zone on ≥3 days/week Anaerobic threshold determination: CPET-based	No	No
11	Koffman et al. (2023) [34]	Wrist-worn	Fitbit Inspire 2	HR zone analysis (descriptive)	Describe time in HR zone, without prescription of a structured training program: Intensity assessment: time spent in PA categories determined based on high/low HR in combination with high/low step count The threshold for high HR was HRrest + 20% HRR, as considered to be the threshold between sedentary and light activity intensity (additional: the high step count threshold was defined as the 25t percentile of the group steps per minute distribution among minutes with at least 1 step recorded (determined equally for all) HRrest determination: mean HR between 2:00 AM and 4:00 AM HRmax determination: with age-dependent formula (220 - age), also for beta-blocker users	Yes	No
12	Schubert et al. (2020) [35]	Wrist-worn	Philips Health Watch (DL8791)	HR zone analysis (descriptive)	Describe time in HR zone, without prescription of a structured training program: Intensity assessment: time spent in moderate intensity zone (=50‐88 bpm above HRrest) HRrest determination: based on low-activity periods (ie, HR when maximum one step per minute and activity count accelerometer <475)	Yes	No
13	Sandberg et al. (2016) [36]	Chest-strap	Actiheart monitor (CamNTech Ltd, Cambridge, UK)	HR zone analysis (descriptive + PA adherence)	Describe time in HR zone, without prescription of a structured training program: Intensity assessment: time spent in moderate to vigorous zone, defined using two methods: (1) HR ≥1.75 * HRrest, and (2) HR ≥ HR value at 3 minutes into a step test (corresponding to 3 METs^f^) PA adherence is defined as: recording at least 150 minutes/week of moderate to vigorous PA (HR ≥ HR value at 3 minutes into a step test), corresponding to 21.4 minutes/day HRrest determination: 30th lowest HR during monitoring period	Yes	No
14	Weeks et al. (2018) [37]	Wrist-worn	Fitbit Charge	HR zone analysis (descriptive + PA adherence)	Describe time in HR zone, without prescription of a structured training program: Intensity assessment: time spent in target HR zone (ie, 55%‐80% of HRmax) and time spent in excessive HR zone (ie,>80% of HRmax) PA adherence is defined as: recording at least 2×10-minutes in target HR zone (ie, 55%‐80% HRmax) on ≥3 days/week (American Heart Association / American Stroke Association (AHA/ASA^g^) guidelines) HRmax determination: with age-dependent Tanaka formula (212‐0.7× age), adjusted for beta-blocker users (Brawner formula: 164‐0.7× age)	No	No
15	Brouwers et al. (2017) [47], SmartCare-CAD	Wrist-worn	MIO Alpha	Physiological estimation using HR-based models (energy expenditure estimation – PAL)	Physical activity level (PAL) is calculated as total energy expenditure (TEE) divided by resting metabolic rate (RMR): TEE^h^ is estimated via multivariate regression using HR above rest (ie, HRnet), accelerometer output (activity counts per minute), weight, age, etc; exact coefficients are not reported RMR^i^ estimated using the Harris-Benedict equation (based on weight, height, age, and sex) PAL thresholds: <3 (light), <6 (moderate), ≥6 (vigorous); typical daily PAL ranges from 1.2 (bed-bound) to >5 (elite athletes)	Yes	No
16	Kraal et al. (2017) [38], FIT@Home	Chest-strap	Garmin FR70	Physiological estimation using HR-based models (energy expenditure estimation – PAL)	Physical activity level (PAL) is calculated as total energy expenditure (TEE) divided by resting metabolic rate (RMR): TEE is estimated via multivariate regression using HR above rest (ie, HRnet), accelerometer output (activity counts per min), weight, age, etc; exact coefficients are not reported RMR estimated using the Harris-Benedict equation (based on weight, height, age, and sex) PAL thresholds: <3 (light), <6 (moderate), ≥6 (vigorous); typical daily PAL ranges from 1.2 (bed-bound) to >5 (elite athletes)	Yes	No
17	Kraal et al. (2016) [39]	Chest-strap	Garmin (only the brand mentioned, not the type)	Physiological estimation using HR-based models (energy expenditure estimation – PAL)	Physical activity level (PAL) is calculated as total energy expenditure (TEE) divided by resting metabolic rate (RMR): TEE is estimated via multivariate regression using HR above rest (ie, HRnet), accelerometer output (activity counts per min), weight, age, etc; exact coefficients are not reported RMR estimated using the Harris-Benedict equation (based on weight, height, age, and sex) PAL thresholds: <3 (light), <6 (moderate), ≥6 (vigorous); typical daily PAL ranges from 1.2 (bed-bound) to >5 (elite athletes)	Yes	No
18	Rissanen et al. (2022) [40], HealthBeat	Chest-strap	Bodyguard 2 (Firstbeat Technologies Oy)	Physiological estimation using HR-based models (VO_2_max^j^ estimation – Firstbeat)	A proprietary algorithm, developed by Firstbeat Technologies Oy, estimates cardiorespiratory fitness levels (VO_2_max in mL/kg/min) The full algorithm and exact details of the procedure are inaccessible, but the algorithm is based on integrating HR (to assess cardiac workload), heart rate variability (to reflect recovery and autonomic function), accelerometer data (to quantify motion and intensity), and individual characteristics (age, sex, weight, height)	Yes	No
19	Sprint et al. (2017) [41]	Wrist-worn	Fitbit Charge	PA change detection	Physical activity change detection (PACD) framework developed to detect changes in PA patterns using HR and step data: Algorithms result in change score (CS^k^) quantifying the magnitude of detected PA changes between time windows Strongest predictors of PA change included time in HR zone (ie, 55%‐80% of HRmax), total step count, and sedentary time reduction HRmax determination: with age-dependent Tanaka formula (212‐0.7× age), adjusted for beta-blocker users (Brawner formula: 164‐0.7× age)	Yes	No
20	Hannan et al. (2021) [42]	Wrist-worn	Lynk2	Personalized activity intelligence (PAI) score calculation	Personal activity intelligence (PAI) is a HR–based metric calculated from exercise intensity and duration: Based on a mathematical algorithm (available in supplementary material of other PAI-publication) It uses HR responses (HRR calculations) over a 7-day rolling window; PAI scores are personalized using age, sex, HRrest, and HRmax HRmax determination: with age-dependent formula (211‐0.64× age), adjusted for beta-blocker or calcium channel blocker users (−15 bpm), or CPET-based when available HRrest determination: not disclosed.	No	No

a HR: heart rate.

b PA: physical activity.

c HRR: heart rate reserve, calculated as HRR = HRmax – HRrest.

d CPET: cardiopulmonary exercise testing.

e Only for intervention group – Polar Verity Sense arm strap for blinded measurements in the control group.

f MET: metabolic equivalent of task.

g AHA/ASA: American Heart Association / American Stroke Association.

h TEE: total energy expenditure.

i RMR: resting metabolic rate.

j VO_2_max: maximal oxygen uptake.

k SC: change score.

### HR-Based Physical Activity Estimation Methods

**Figure 2.**
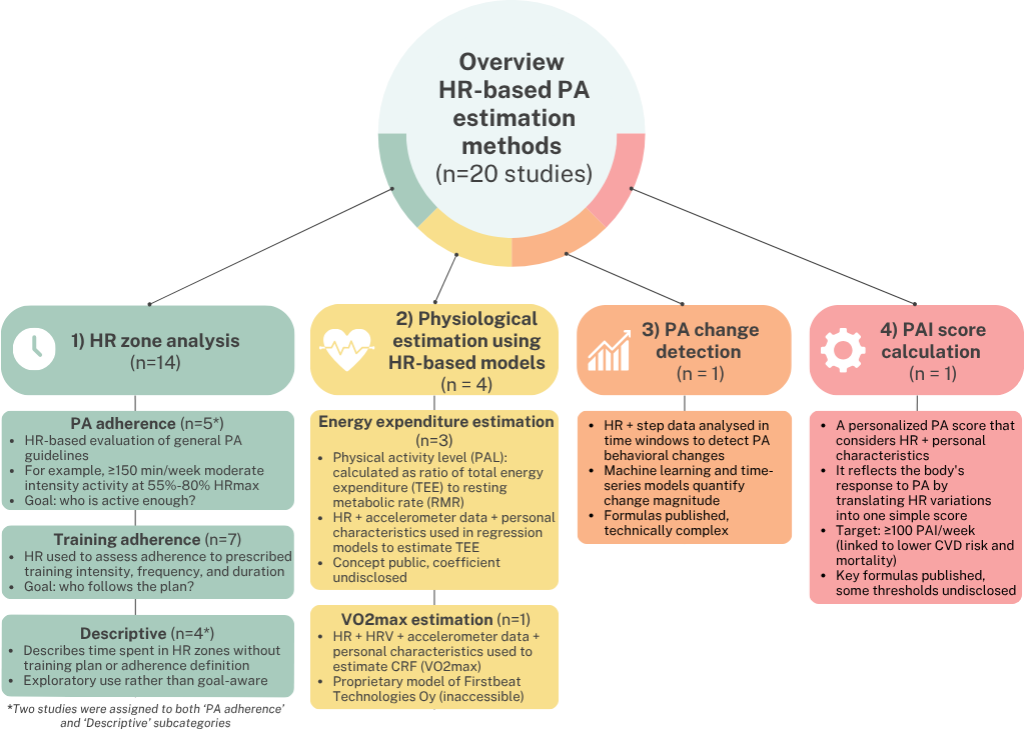
Overview of heart rate-based physical activity estimation methods identified in this review. CRF: cardiorespiratory fitness; CVD: cardiovascular disease; HR: heart rate; HRmax: maximal heart rate; HRV: heart rate variability; PA: physical activity; PAI: personal activity intelligence; PAL: physical activity level; RMR: resting metabolic rate; TEE: total energy expenditure; VO_2_max: maximal oxygen uptake.

Four distinct approaches to HR-based PA estimation were identified: (1) HR zone analysis, (2) physiological estimation using HR-based models, (3) PA change detection, and (4) personalized activity scoring. These methods vary in complexity, ranging from simple threshold-based evaluations to algorithmic pattern detection. Each approach is summarized in Figure 2 and further elaborated in the subsections below, while detailed study specifications are presented in Table 2 (PA estimation approach) and in Table S2 in Multimedia Appendix 1.

#### HR Zone Analysis

Across 14 of the 20 included studies [28-37,43-46], the time spent in predefined HR zones was used to evaluate PA intensity and/or adherence, either in relation to general PA recommendations, structured training goals, or purely descriptively. These approaches relied on rule-based threshold classifications rather than analytical or statistical modeling, with HR values categorized into predefined HR-based intensity zones to quantify time spent at different activity levels.

HR zones were typically defined using heart rate reserve (HRR=HRmax [maximal heart rate]–HRrest) or percentages of HRmax. HRmax was determined using age-based formulas (n=4) [31,34,37,44], sometimes corrected for beta blocker use (n=1) [37], or measured via cardiopulmonary exercise testing (CPET; n=6) [28-30,32,45,46]. HRrest was estimated using self-established definitions such as the 30th lowest HR during monitoring, the average HR between 2:00 AM and 4:00 AM, or HR observed during low-activity periods (eg, HR when <1 step/minute combined with an accelerometer count <475). Most studies relied on transparent zone definitions, and most methods were reproducible. A subdivision was made for studies that applied HR zones as (1) behavioral benchmarks (eg, to assess adherence to PA guidelines), (2) criteria for training adherence, or (3) exploratory tools to describe PA intensity.

1. PA adherence: Around 5 [28,36,37,43,44] studies assessed whether participants met general PA recommendations based on HR-derived intensity thresholds. The internationally accepted target of ≥150 minutes per week of moderate-intensity activity was typically applied, or translated into a daily equivalent (ie, ≥21.4 minutes/day). Moderate intensity was typically defined as 40%‐70% or 40%‐80% of HRR, or 55%‐80% of HRmax. One study defined the target threshold more pragmatically, using the HR at minute 3 of a step test as the intensity cut-off. In some cases, the HR-derived method of checking PA adherence was compared with assessments based on accelerometer-based indicators (n=2 [36,43]; eg, total accelerometer counts) and/or PA questionnaires (n=3 [28,43,44]; eg, report ≥150 minutes of moderate and/or vigorous intensity activity on a PA questionnaire, like the physical activity scale for individuals with physical disabilities [PASIPD]).
2. Training adherence: A total of 7 [29-33,45,46] studies evaluated adherence to a structured training program using HR to monitor both exercise intensity and completion. These programs defined frequency, duration, and intensity in advance (eg, 3×/week, 30‐60 min/session, 70%‐80% HRR). Qualitative adherence was assessed based on intensity, including time spent in the prescribed HR zone, average session intensity (eg, 74.8% of HRR), or retention ratios (eg, percentage of total exercise time in the zone). Quantitative adherence referred to session completion, often defined as attending ≥70% of prescribed sessions, or meeting minimum thresholds such as ≥10 minutes/day in the target zone on ≥3 days/week.
3. Descriptive: In 4 [34-37] studies, HR zones were used without a behavioral target or structured plan. These studies described the time spent in various HR zones to characterize cardiovascular load or exercise intensity without defining adherence. The aim was typically exploratory, offering insight into physiological patterns of activity.

Feedback mechanisms: Among the 14 studies using HR zone analysis, feedback strategies varied in intensity and purpose. Four [30-33] training adherence studies delivered real-time feedback via wearable devices (eg, LED color changes or vibrations) when HR deviated from target zones. In at least 6 [28,29,43-46] studies, HR data were reviewed by health professionals via dashboards or apps (eg, Polar Flow; Polar Electro), with individual (telephone-based) or group-based counseling provided with a focus on knowledge, attitudes, self-efficacy, and self-management skills, and often informed by behavior change and motivational interviewing techniques. The remaining 4 [34-37] studies used HR monitoring purely for retrospective analysis, without participant-facing feedback or behavioral guidance.

#### Physiological Estimation Using HR-Based Models

A total of 4 studies [38-40,47] used HR data to estimate derived physiological PA-related outcomes rather than to assess PA behavior and adherence. These outcomes included energy expenditure, expressed as physical activity level (PAL), and cardiorespiratory fitness (CRF), typically estimated as VO_2_max. In both cases, HR was used as a physiological input parameter, often in combination with accelerometer data and individual characteristics to inform regression or algorithmic models.

1. Energy expenditure–PAL: A total of 3 [38,39,47] studies estimated daily energy expenditure by calculating PAL, defined as the ratio of total energy expenditure (TEE) to resting metabolic rate. TEE was estimated using multivariate regression models that included HR above rest, accelerometer-derived activity counts per minute, and participant characteristics such as age and weight. The resting metabolic rate was estimated using the Harris-Benedict equation, based on sex, weight, height, and age. While none of the studies disclosed the exact regression coefficients or intercepts used to model TEE, the overall structure of the approach was well described and reproducible in principle. PAL was used as a continuous indicator of daily activity level, and typical values range from 1.2 (bed-bound) to above 5.0 (elite athletes). In these studies, no behavioral adherence thresholds or real-time feedback mechanisms were applied to PAL scores, though retrospective comparisons were made between groups (eg, cardiac telerehabilitation vs center-based programs).
2. VO_2_max estimation–firstbeat: One study by Rissanen et al [40] estimated CRF by calculating VO_2_max using a wearable device during a 30-minute self-paced walk. The algorithm integrated HR, heart rate variability, and movement data, alongside participant characteristics (eg, age, sex, height, weight, and HRmax), to model aerobic capacity. The HRmax determination was not disclosed. The estimation was validated against CPET-derived VO_2_max values. The model showed strong agreement, with a mean absolute error of 3.1 mL/kg/min, mean absolute percentage error of 10.4%, and intraclass correlation coefficient of 0.88. The study acknowledged reduced accuracy in subgroups such as patients with type 2 diabetes. Developed by Firstbeat Technologies Oy, the algorithm is proprietary, with only conceptual elements publicly available. It remains inaccessible and is commercially protected, limiting reproducibility and external validation.

#### Physical Activity Change Detection

The study by Sprint et al [41] introduced the Physical Activity Change Detection (PACD) framework, an analytical method designed to identify significant changes in PA behavior over time using HR and step count data. PACD applied time-series analysis to detect meaningful fluctuations in PA intensity, frequency, and duration.

The dataset was segmented into consecutive time windows (eg, per week). Pairs of these windows were compared using three algorithmic methods: (1) small window permutation-based change detection (sw-PCAR), which uses Kullback-Leibler divergence to measure differences in activity patterns between two time windows, with significance assessed through a permutation test. (2) distance-based dissimilarity scorer (DDS), which computes the average Euclidean distance between summary statistics (eg, steps/day, time in HR zones), with distances above 0.25 interpreted as meaningful deviations. (3) Virtual classifier (VC), a machine learning method (decision trees) trained to distinguish activity patterns between windows, using classifier accuracy as a measure of behavioral change. Key predictors of PA change were fewer bouts in the target HR zone (55%‐80% HRmax), reduced sedentary time (<5 steps/minute), and lower daily step counts.

Each algorithm produced a change score reflecting the magnitude of PA change between time windows. In this study, PACD was applied retrospectively to characterize PA changes during inpatient rehabilitation, without being used for real-time clinical decision-making. Although no open-source code was provided, the authors detailed the full methodology, including formulas and statistical techniques, enabling potential reproduction of the analysis by researchers with appropriate technical expertise.

#### Personal Activity Intelligence (PAI) Score Calculation

One study by Hannan et al [42] used HR data to calculate the Personal Activity Intelligence (PAI) score, a metric that transforms HR data from a wearable device into a personalized, feedback score for long-term health maintenance. The PAI algorithm integrates HR intensity and duration over a rolling 7-day period and accounts for individual characteristics such as age, sex, HRrest, and HRmax [48]. The goal is to provide a single number reflecting whether an individual maintains sufficient cardiovascular stimulus to reduce morbidity and mortality risk [49]. A weekly score of ≥100 PAI is considered optimal for health benefits based on prior retrospective longitudinal cohort studies [50,51].

In the study of Hannan et al [42], PAI was used in a 2-phase design. Participants initially wore a HR monitor in a blinded phase (without receiving feedback), followed by an unblinded phase where they had access to their PAI score and were encouraged to increase it. HRmax, needed for the PAI calculation, was estimated using an age-dependent formula (211‐0.64 × age), with correction for beta- or calcium-channel blocker use (–15 bpm), or derived via CPET when available. The authors reported that 89% of participants improved their total PAI score following access to real-time feedback. The proportion of individuals achieving ≥50 PAI per week increased from 39% to 61% across phases [42].

Key components and equations behind the PAI algorithm, originally developed by the Cardiac Exercise Research Group (CERG) at the Norwegian University of Science and Technology (NTNU), have been published in supplementary material of earlier PAI-studies [18], although these were not eligible for inclusion in this review due to their retrospective, questionnaire-based methodology and noncardiac populations (see Discussion). The model is therefore largely reproducible for researchers with sufficient technical expertise, though some implementation details (eg, thresholds to begin earning PAI) remain undisclosed.

### Methodological Quality Assessment

The CCAT quality ratings ranged from 50% to 87%, with a mean of 71% (Table 3). Four studies scored above 75%, indicating high methodological quality, while 2 [26,27] studies scored below 60%, reflecting some limitations. The remaining 14 studies fell within the moderate range of 60%-75%. Most studies performed well in the domains of introduction, data collection, and discussion, with the majority scoring between 4 and 5 in these areas and no study scoring below 3. Fifteen studies [28-30,32-37,39-43,47] reached a moderate score of 3 in the design domain. Sampling and ethics were the weakest domains; 5 studies [30-32,39,45] scored below 3 for sampling, and 3 studies [30-32] scored below 3 for ethics, highlighting issues with sample representativeness and ethical reporting.

**Table 3.** Quality rating using CCAT^a^. The methodological quality of the included studies was assessed using the CCAT [26,27], which evaluates eight domains: Preliminaries, Introduction, Design, Sampling, Data collection, Ethics, Results, and Discussion. Each domain is scored from 0 to 5, with a maximum total score of 40. For protocol descriptions, only applicable domains were scored. Domains not applicable due to the absence of empirical data (eg, Results and Discussion) were marked as not Applicable (N/A). The total score for protocols was recalculated as a percentage of the maximum applicable score (eg, if 6 domains were scored, the total score was calculated out of 30).

ID	Study	CCAT^a^ rating per domain									
		1	2	3	4	5	6	7	8		
		Preliminaries (0‐5)	Introduction (0‐5)	Design (0‐5)	Sampling (0‐5)	Data collection (0‐5)	Ethics (0‐5)	Results (0‐5)	Discussion (0‐5)	Total score	Total percentage (%)
1	Alonso et al (2021) [28] *HEART Camp*	3	4	3	3	3	5	4	4	29/40	73
2	Crozier et al (2023) [43] *MOTIVATE-CR+*	4	4	3	3	3	4	—^b^	—	21/30	70
3	Mansfield et al (2017) [44] *PROPEL*	4	5	4	4	4	5	—	—	26/30	87
4	Batalik et al. (2020) [29]	4	4	3	3	4	4	3	4	29/40	73
5	Chung et al (2019) [30]	2	4	3	2	4	2	3	3	23/40	58
6	Dobrican and Zampunieris (2016) [31]	3	3	2	2	3	2	2	3	20/40	50
7	Dosbaba et al (2020) [45]	3	3	4	2	3	4	—	—	19/30	63
8	Quero et al (2017) [32]	3	4	3	2	4	2	3	3	24/40	60
9	Suchy et al (2014) [46] *OptimEx-CLIN*	4	4	4	4	3	4	—	—	23/30	77
10	Xia et al (2023) [33]	4	4	3	3	4	3	4	4	29/40	73
11	Koffman et al (2023) [34]	4	5	3	4	4	4	4	4	32/40	80
12	Schubert et al (2020) [35]	3	3	3	3	4	4	4	4	28/40	70
13	Sandberg et al (2016) [36]	3	4	3	4	4	4	4	3	29/40	73
14	Weeks et al (2018) [37]	4	4	3	3	4	4	3	4	29/40	73
15	Brouwers et al (2017) [47] *SmartCare-CAD*	3	4	3	3	3	4	—	—	20/30	67
16	Kraal et al (2017) [38] *FIT@Home*	4	5	4	3	3	4	4	4	31/40	78
17	Kraal et al (2016) [39]	4	4	3	2	4	4	4	4	29/40	73
18	Rissanen et al. (2022) [40] *HealthBeat*	4	4	3	3	3	4	4	4	29/40	73
19	Sprint et al. (2017) [41]	4	4	3	3	4	4	3	4	29/40	73
20	Hannan et al. (2021) [42]	4	4	3	3	3	4	4	4	29/40	73

a CCAT: Crowe Critical Appraisal Tool.

b not applicable

The methodological quality assessment was used to contextualize the interpretation of findings but did not determine study inclusion. Importantly, studies with lower scores were distributed across the different methodological categories identified in the narrative synthesis, and the methodological approaches used to derive HR-based PA metrics were largely consistent across studies irrespective of CCAT score. Overall, the quality of the included studies was moderate to high.

## Discussion

### Principal Findings

This review provides the first structured overview of HR-based methodologies for PA estimation in patients with CVD, highlighting their clinical utility, strengths, and the implementation gaps. We identified 4 distinct estimation approaches: HR zone analysis, physiological estimation using HR-based models, PA change detection, and personal activity intelligence score calculation. Most studies (14/20) used HR zone thresholds to evaluate PA intensity or adherence, often within CR contexts. Other methods incorporated algorithmic modeling or machine learning to derive metrics such as energy expenditure, CRF, or behavioral change. While these approaches varied in complexity and purpose, each demonstrated the potential of HR data to provide physiologically meaningful insight into daily PA patterns.

### HR Zone Analysis and Guideline-Based Adherence

HR zone analysis emerged as the most commonly applied and straightforward method of HR-based PA estimation in this review. It is straightforward because it allows for a direct translation of international PA guidelines into quantifiable metrics, specifically, the time spent in moderate-to-vigorous intensity HR zones, and it aligns with the widely adopted the frequency, intensity, time, and type framework [1,4]. As such, it provides clinicians and researchers with an accessible method to both prescribe and evaluate PA intensity in line with global recommendations.

Both the World Health Organization (WHO) and the European Society of Cardiology (ESC) advise that adults should engage in at least 150 minutes of moderate or 75 minutes of vigorous intensity aerobic activity per week, or an equivalent combination of both, with intensity defined via several physiological indicators, including %VO_2_max, %HRmax, %HRR, or ratings of perceived exertion [1,4]. Among the reviewed studies, %HRmax and %HRR were used most often to define intensity zones. ESC recommends that maximal HR values should ideally be based on exercise testing rather than estimated from age-predicted formulas, especially in clinical populations and in patients on rate-limiting medications such as beta-blockers [1]. Despite this, most studies in our review relied on simple prediction equations for HRmax, which may result in less precise zone definitions. Notably, none of the included studies explicitly reported whether vigorous-intensity minutes were counted double when evaluating weekly MVPA totals. While international guidelines do allow for this (eg, 75 min of vigorous activity or an equivalent combination with moderate activity) [1,4], the absence of clear reporting may limit interpretability and cross-study comparability. Including this distinction in future reporting could help ensure more accurate and standardized assessments of adherence.

HRrest, another component needed for calculating HRR, was often defined through study-specific approaches, such as averaging nighttime HR values or identifying low-HR periods during inactivity. While literature suggests that early morning supine HR, measured immediately after waking but before getting out of bed, may offer the most reliable estimate, there is no standardized or transparent method for deriving HRrest from wearable data at the time of this writing [52]. Most commercial devices provide HRrest estimates through proprietary algorithms, leaving researchers with limited insight into how these values are calculated.

In terms of adherence assessment, some studies focused on guideline-based targets (eg, ≥150 min/week of MVPA), while others evaluated compliance with structured training programs based on the frequency, intensity, time, and type principles. Importantly, HR monitoring enabled a distinction between session attendance (frequency) and session quality (intensity and duration), offering a more nuanced understanding of adherence by distinguishing between mere participation and actual achievement of intensity and duration targets [53].

Finally, several studies integrated HR zone data into feedback mechanisms, either in real time (eg, via smartwatches with LED zone indicators) or post-session through digital dashboards and clinician-guided counseling. Effective feedback has been shown to be a key driver of behavior change in PA interventions [54]. When combined with motivational strategies such as self-monitoring and goal setting, these feedback loops may support greater behavioral change and patient engagement toward sustained PA adherence [43,44,54].

### HR Versus Accelerometer-Based PA Monitoring

Several studies in this review combined HR monitoring with accelerometer-based methods to quantify PA adherence and estimate physiological parameters such as energy expenditure and VO_2_max. These multimodal approaches aimed to improve accuracy and/or cross-validate findings by combining the complementary strengths of each method [55]. HR monitoring provides a direct physiological measure of exercise intensity and cardiovascular load, making it a more personalized and clinically relevant indicator of effort than mechanical metrics such as step count or accelerometer counts. Moreover, the physical demand associated with a given accelerometer value can vary substantially between individuals depending on their fitness, age, or health status [56]. For example, walking at the same pace may represent light intensity for one person and moderate intensity for another [36].

Despite these limitations, accelerometer and step count data could play a valuable role in assessing sedentary time, an increasingly recognized factor in cardiovascular health [57]. Sedentary behavior, defined as waking behavior in a sitting, reclining, or lying posture with low energy expenditure, has been associated with cardiometabolic risk, even in individuals who meet weekly MVPA guidelines [58,59]. This risk profile may reflect distinct physiological pathways, underscoring the importance of addressing sedentary behavior alongside PA adherence [60]. In the PACD study included in this review, a reduction in sedentary time (defined as time with fewer than 5 steps per minute) emerged as a key predictor of meaningful PA behavior change [41]. When paired with HR data to reflect oxygen demand and PA intensity, step-based indicators of inactivity may offer a low-burden, scalable method for tracking both sedentary time and physiological load. However, it is important to assess PA and SB as independent behavioral domains, each with distinct health effects and potentially requiring their own intervention strategies [60].

### From Activity Monitoring to Physiological Outcomes

Although most studies focused on measuring adherence to PA guidelines (eg, time in zones), a smaller subset advanced toward estimating physiological outcomes from HR data, such as energy expenditure (PAL) or cardiorespiratory fitness (VO_2_max). This transition from behavioral metrics to physiological modeling represents a valuable next step, especially in clinical populations where improving aerobic capacity and reducing cardiovascular risk are primary goals.

For example, three studies estimated PAL, defined as the ratio of total energy expenditure to resting metabolic rate. These models typically combined HR above rest with accelerometer data and participant characteristics [38,39,47]. PAL is widely used in nutrition science to estimate daily energy needs, but in cardiovascular care its clinical application remains limited [61]. As a descriptive metric, it lacks behavioral thresholds, is rarely linked to feedback mechanisms, and often relies on regression models with undisclosed coefficients, limiting transparency and reproducibility in research and practice. A more advanced example is the proprietary Firstbeat algorithm, which estimated VO_2_max using HR, HR variability, movement data, and participant characteristics during a 30-minute walk [40]. While generalizability to cardiac populations remains uncertain and requires further validation, the Firstbeat algorithm showed acceptable agreement with CPET-derived values in athletic cohorts or adults with cardiovascular risk factors and is now embedded in commercial wearables from Garmin, following their acquisition of Firstbeat Technologies Oy in 2020 [40,62,63]. While this integration supports scalability, the proprietary nature of the algorithm limits its adaptability and full validation in diverse patient groups.

Together, these examples illustrate a growing shift from pure activity tracking toward predictive modeling of physiological capacity. By combining HR data with individual and contextual factors, such models could move beyond surface-level monitoring to support personalized CR planning and long-term health surveillance. Such approaches may help clinicians and researchers estimate meaningful outcomes, such as changes in cardiorespiratory fitness, based on daily-life activity patterns, ultimately linking daily PA behavior to cardiovascular impact.

### PAI: Validation, Challenges, and Future Directions

The PAI system stands out within this review as the only approach that translates continuous HR data into a personalized, interpretable single-number feedback metric intended to motivate behavioral change over time. PAI reflects accumulated cardiovascular effort across a rolling 7-day window. In the one included PAI study in this review, short-term improvements in PAI accumulation were observed after CR participants gained access to their PAI score via a smartphone application, although evidence remains exploratory [42].

As a caveat, the commonly cited ≥100 PAI/week threshold, proposed to reduce the risk of CVD and mortality, originates from retrospective analyses of large population studies (eg, the HUNT Study in Norway) [48,51,64-68]. During the screening process of this review, eight such validation studies were identified but excluded, as none used HR monitors to calculate PAI [48,51,64-68]. Instead, PAI scores were estimated post hoc from self-reported PA questionnaires, relying on participants’ recall of activity frequency, intensity, and duration [48,51,64-68]. This introduces substantial subjectivity and deviates from the intended physiological, HR-based calculation of PAI, potentially reducing both accuracy and internal validity.

Moreover, 7 of the 8 validation studies were conducted in healthy adult populations, frequently excluding participants with cardiovascular conditions. The reported mean ages were relatively young, ranging from 42.6 [64] to 55.1 [68] years. One study included participants with self-reported angina pectoris, myocardial infarction, or stroke, with a higher mean [SD] age of 67.6 [10.3] years[66]. However, activity in that group was still assessed via questionnaires, and the population was not clinically verified. To date, no validation studies have assessed the accuracy or clinical utility of PAI when calculated from real HR data in older adults or patients with CVD, populations for whom accumulating ≥100 PAI points per week may be less achievable but important. Also, the PAI algorithm rewards sustained vigorous activity more than moderate or lower-intensity efforts, limiting its applicability for clinical populations where activities such as walking or cycling are more prevalent [64]. A summary table of the characteristics of the 8 excluded PAI validation studies is available in Table S3 in Multimedia Appendix 1.

Although the algorithm is largely reproducible based on published formulas, certain implementation details remain proprietary [18]. Researchers with technical expertise can replicate the model in principle, but public tools for doing so are limited. The MIA Health app, currently the only available platform for real-time PAI calculation, expanded its availability in January 2025 from a few Nordic countries to most of Europe, North America, Australia, New Zealand, Taiwan, and Brazil, potentially increasing both clinical and consumer access [69].

Recently, a follow-up metric known as the activity quotient (AQ) was introduced by the same research group as a refinement of the PAI. Some retrospective studies have validated AQ using the same questionnaire-based methodology as PAI and report stronger associations with VO_2_max and mortality [70]. However, none of these studies used HR monitors, and prospective AQ studies with wearable devices are still forthcoming [69]. In parallel to these developments, our research group is developing the Antwerp Activity Index and smart Antwerp Activity Index models [71,72] (P Vermunicht, PhD, unpublished data, 2026). These tools aim to provide HR-based, personalized activity scores by integrating daily-life HR data with individual characteristics, and are designed to predict cardiorespiratory fitness changes over time. While preliminary development has been promising, full clinical validation and prospective outcome studies are currently underway. Such emerging models may further advance the translation of HR-derived activity monitoring into actionable, personalized health guidance in cardiac populations.

Together, these developments reflect a growing interest in HR-based PA quantification and behavioral feedback. However, their clinical relevance for cardiac and older adults will depend on prospective validation in target populations, improved algorithmic transparency, and appropriate feedback tailoring for diverse activity profiles.

### Future Directions and Research Limitations

The reviewed studies illustrate a diverse range of HR-based methods, from guideline-driven time-in-zone metrics to more advanced models estimating physiological outcomes and personal activity scores. This variety reflects a growing interest in using HR data not only to monitor activity but also to estimate its cardiovascular implications. Future research should prioritize validation of these methods in real-world populations with CVD, with attention to feasibility, clinical interpretability, and predictive value. Tools that go beyond simple time-in-zone tracking and instead estimate outcomes like energy expenditure or changes in cardiorespiratory fitness may offer greater clinical value. As illustrated by systems such as PAI or the Firstbeat VO_2_max model, combining HR data with individual characteristics can generate personalized feedback with the potential to guide long-term health behavior. Future work should strive for a balance between algorithmic sophistication, transparency, and accessibility.

Although not a focus of this review, a key translational consideration for HR-based PA estimation is that the validity of any derived PA metric depends on the accuracy of the underlying HR data [73,74]. Although ECG-based chest straps provide highly accurate HR measurements, they are less practical for daily monitoring. Wrist-worn photoplethysmography devices are more convenient, but their accuracy can be affected by artifacts and they should be validated against an ECG before clinical use [75,76]. Recent work from our group has therefore focused on artifact detection and cleaning procedures that operate directly on device-generated photoplethysmography HR data from commercial wearables, aiming to remove unreliable data while preserving clinically meaningful activity-related HR fluctuations [71,76-78]. Such artifact removal strategies are highly relevant when implementing the HR-based PA quantification methods described in this review, particularly in real-world cardiac populations where pathophysiological factors such as peripheral edema, poor tissue perfusion, and a higher incidence of arrhythmias may affect HR reliability [76,79].

Beyond methodological development, successful implementation of HR-based PA estimation also depends on how these metrics are embedded within broader digital care strategies. Mobile health tools that support patient education, self-management, and structured follow-up may enhance engagement and facilitate behavior change, thereby increasing the clinical relevance of HR-derived activity metrics [80,81].

This review has several limitations. It was not prospectively registered in a public database (eg, PROSPERO [International Prospective Register of Systematic Reviews]), which may limit transparency regarding protocol deviations. Although a protocol was developed to guide the review process, it was not publicly available. As a narrative synthesis, it does not provide pooled effect sizes or meta-analytic comparisons. The included studies varied widely in design, sample size, and how HR-based metrics were operationalized. While this limits direct comparability, the primary aim of this review was to map the diversity of methodological approaches rather than pool results. In addition, studies using techniques such as PACD or personalized scoring systems remain scarce, restricting broader generalization. Continued development and validation of such tools, especially in older and cardiac populations, will be essential to support their integration into clinical practice. Finally, due to the rapid evolution of wearable technologies and increasing research interest in HR-based activity estimation, new studies may have been published after the final search date. For instance, an eligible outcome paper from the OptimEx-Clin trial was published shortly after the search period [82]. While this study was not included for methodological consistency, its design was captured through the previously published protocol paper, which is part of this review. Future updates of this review could consider incorporating such newly available outcome data.

82

### Conclusions

This review provides the first structured synthesis of HR-based methodologies for PA estimation in patients with CVD. Most studies relied on HR zone analysis to evaluate adherence to international PA guidelines, while others explored more advanced modeling approaches to estimate physiological outcomes such as energy expenditure or cardiorespiratory fitness.

Our findings underscore that HR monitoring offers a physiologically meaningful and scalable way to estimate PA, especially in clinical populations where traditional step-based or questionnaire-based methods may fall short. Personalized HR-based feedback systems may offer intuitive activity targets for patients. However, variability in how HR metrics are defined, validated, interpreted, and applied, currently limits comparability and implementation. Future research should focus on validating HR-based methods in real-world populations with CVD, improving methodological transparency, and tailoring feedback to individual needs. Ensuring reliable HR measurements remains a prerequisite for meaningful HR-based PA estimation in clinical practice. As wearable technologies continue to advance, HR-derived metrics hold strong potential to support more personalized and effective PA guidance in both clinical care and daily life.

## Supplementary material

10.2196/79995 Multimedia Appendix 1 Supporting information.

10.2196/79995 Checklist 1 PRISMA 2020 checklist.

## Abbreviations

ACS: acute coronary syndrome
AQ: activity quotient
CABG: Coronary artery bypass grafting
CAD: coronary artery disease
CCAT: Crowe Critical Appraisal Tool
CENTRAL: Cochrane Central Register of Controlled Trials
CERG: Cardiac Exercise Research Group
CPET: cardiopulmonary exercise test
CR: cardiac rehabilitation
CRF: cardiorespiratory fitness
CVD: cardiovascular disease
ECG: electrocardiogram
ESC: European Society of Cardiology
HFpEF: heart failure with preserved ejection fraction
HFrEF: heart failure with reduced ejection fraction
HR: heart rate
HRR: heart rate reserve
MVPA: moderate-to-vigorous physical activity
NTNU: Norwegian University of Science and Technology
PA: physical activity
PACD: Physical Activity Change Detection
PAI: Personal Activity Intelligence
PAL: physical activity level
PCI: percutaneous coronary intervention
PROSPERO: International Prospective Register of Systematic Reviews
RCT: randomized controlled trial
TEE: total energy expenditure
VO₂max: maximal oxygen uptake
WHO: World Health Organization
