# Supplementary material for: Heart Rate Monitors for the Estimation of Physical Activity in Patients With Cardiovascular Disease: Systematic Review
Source: JMIR Mhealth Uhealth. 2026 Jun 17;14:e79995. doi: 10.2196/79995 (PMC13274969; doi:10.2196/79995)
Supplement: Multimedia Appendix 1 [file mhealth-v14-e79995-s001.pdf]

Multimedia Appendix – Supporting Information

Table S1. Search strategy.

Database	Search	Query	Items found
PubMed	#1	"Heart Diseases"[Mesh] OR "Heart Disease*" OR "Cardiac Disease*" OR "Cardiovascular Disease*" OR "Cardiac Disorder*" OR "Heart Disorder*" OR "Cardiac Rehabilitation"[Mesh] OR Cardiac Rehabilitation* OR Cardiovascular Rehabilitation* OR "Cardiac Patient*" OR "Heart Patient*" OR "Cardiovascular Patient*" OR Cardiac Telerehabilitation*	1,692,701
	#2	"Heart Rate"[Mesh] OR Heart Rate* OR "Cardiac Rate*" OR "Pulse Rate*" OR "Heartbeat*" OR "Heart Rate Determination"[Mesh] OR "Heart Rate Determination*" OR "Heart Rate Monitoring"	476,114
	#3	"Fitness Trackers"[Mesh] OR Fitness Tracker* OR "Tracker*" OR "Physical Fitness Tracker*" OR "Activity Tracker*" OR "Personal Fitness tracker*" OR "Wearable Electronic Devices"[Mesh] OR Wearable Electronic Device* OR "Wearable Technology" OR "Wearable Device*" OR "Activity Monitor" OR "Physical Activity Monitor" OR "Wearable" OR "Smartwatch" OR "Chest Strap" OR "Chest Band" OR "Heart Rate Monitor*" OR "Heart Rate Device*" OR "Wearable Device*"	53,121
	#4	"Exercise"[Mesh] OR "Exercise*" OR "Physical Activit*" OR "Activit*" OR "Physical Exercise*" OR "Aerobic Exercise*" OR "Exercise Training*" OR "Physical Activity Tracking"	4,332,478
	#5	#1 AND #2 AND #3 and #4	524
	#6	#1 AND #2 AND #3 and #4 Filters: in the last 10 years	405
Web of Science	#1	ALL=("Heart Disease*" OR "Cardiac Disease*" OR "Cardiovascular Disease*" OR "Cardiac Disorder*" OR "Heart Disorder*" OR "Cardiac Rehabilitation*" OR Cardiovascular Rehabilitation* OR "Cardiac Patient*" OR "Heart Patient*" OR "Cardiovascular Patient*" OR Cardiac Telerehabilitation*)	606,083
	#2	ALL=("Heart Rate*" OR "Cardiac Rate*" OR "Pulse Rate*" OR "Heartbeat*" OR "Heart Rate Determination*" OR "Heart Rate Monitoring")	215,199

	#3	ALL=("Fitness Tracker*" OR "Tracker*" OR "Physical Fitness Tracker*" OR "Activity Tracker*" OR "Personal Fitness tracker*" OR Wearable Electronic Devices* OR "Wearable Technology" OR "Wearable Device*" OR "Activity Monitor" OR "Physical Activity Monitor" OR "Wearable" OR "Smartwatch*" OR "Chest Strap*" OR "Chest Band*" OR "Heart Rate Monitor*" OR "Heart Rate Device*" OR "Wearable Device*")	128,415
	#4	ALL=("Exercise*" OR "Physical Activit*" OR "Activit*" OR "Physical Exercise*" OR "Aerobic Exercise*" OR "Exercise Training*" OR "Physical Activity Tracking")	6,079,704
	#5	#1 AND #2 AND #3 and #4	309
	#6	#1 AND #2 AND #3 and #4 and 2024 or 2023 or 2022 or 2021 or 2020 or 2019 or 2018 or 2017 or 2016 or 2015 or 2014 (Publication Years)	247
CENTRAL	#1	("Heart Disease*" or "Cardiac Disease*" or "Cardiovascular Disease*" or "Cardiac Disorder*" or "Heart Disorder*" or "Cardiac Rehabilitation*" or Cardiovascular Rehabilitation* or "Cardiac Patient*" or "Heart Patient*" or "Cardiovascular Patient*" or Cardiac Telerehabilitation*).af.	197,302
	#2	("Heart Rate*" OR "Cardiac Rate*" OR "Pulse Rate*" OR "Heartbeat*" OR "Heart Rate Determination*" OR "Heart Rate Monitoring").af.	81,176
	#3	("Fitness Tracker*" OR "Tracker*" OR "Physical Fitness Tracker*" OR "Activity Tracker*" OR "Personal Fitness tracker*" OR Wearable Electronic Devices* OR "Wearable Technology" OR "Wearable Device*" OR "Activity Monitor" OR "Physical Activity Monitor" OR "Wearable" OR "Smartwatch*" OR "Chest Strap*" OR "Chest Band*" OR "Heart Rate Monitor*" OR "Heart Rate Device*" OR "Wearable Device*").af.	5,964
	#4	("Exercise*" OR "Physical Activit*" OR "Activit*" OR "Physical Exercise*" OR "Aerobic Exercise*" OR "Exercise Training*" OR "Physical Activity Tracking").af.	338,137
	#5	#1 AND #2 AND #3 and #4	229
	#6	Limit #5 to yr="2014-Current"	171

The searches were conducted on April 3, 2024. The search strategies were developed in English and applied without language filters at the database level; however, only full-text articles published in English were included during screening, as specified in the eligibility criteria.

Table S2. Study aims, intervention/observation periods, outcomes and main findings.

Study ID	First author (year)	Aim study	Description of the intervention or observation period	Duration of the intervention or observation period	Description of the control group	Outcomes	Main findings
1	**Alonso et al. (2021)** [28] *HEART Camp*	To evaluate the HEART Camp intervention on adherence with exercise recommendations in the HFpEF and HFrEF subgroups	• Access to an exercise facility + PA coaching using both group-based and individual-based strategies<br>• Focus on knowledge, attitudes, self-efficacy, behavioral self-management skills and social support<br>• Based on principles of behavior change	18 months	Enhanced usual care: standard care + access to an exercise facility	• Amount of patients adherent to PA guidelines<br>• Objective physical function (6-Minute Walk Test) and patient-reported health status (Kansas City Cardiomyopathy Questionnaire), anxiety, depression, role satisfaction, fatigue, pain interference, physical function, and sleep disturbance	• **PA adherence: A) HFpEF**: Adherence was higher in the intervention group at 12 months (42% vs. 14%) and 18 months (56% vs. 0%), but not significantly different at 6 months (42% vs. 19%); **B) HFrEF**: Adherence in the intervention group (36%, 42%, 29%) was similar to the control group (38%, 34%, 29%) at all time points<br>• **HEART Camp intervention**: improved long-term adherence to exercise, physical function, and patient-reported outcomes in HFpEF, and reduced anxiety in HFrEF
2	**Crozier et al. (2023)** [29] *MOTIVATE-CR+*	To evaluate the feasibility of an mHealth-driven home walking program immediately posthospital discharge to increase uptake to CR	• mHealth-driven home walking program prescribed by exercise specialists, including four counselling sessions<br>• Before and during center-based CR<br>• Based on principles of behavior change<br>• App for patients (Polar Flow), dashboard for exercise specialists (Polar Flow for coach)	>3 months	Standard center-based CR	• Intervention feasibility: screening, eligibility, participation rates, CR uptake, dropout reasons, and outcome assessment completion<br>• Exercise adherence & outcomes: Godin-Shephard Leisure-Time Physical Activity Questionnaire, objective activity levels, body composition, height, weight, waist circumference, blood pressure, blood lipids<br>• Health & patient feedback: quality of life (EQ-5D), healthcare usage, and post-intervention acceptability (interviews)	NA
3	**Mansfield et al. (2017)** [30] *PROPEL*	To evaluate if an exercise and self-management intervention (PROPEL) can increase participation in PA after stroke rehabilitation	• Weekly group discussions on PA maintenance<br>• Based on principles of behavior change<br>• Specific attention is paid on how to monitor HR-based exercise intensity<br>• In addition to a group aerobic exercise program	6 weeks	Group aerobic exercise program	• Amount of patients adherent to PA guidelines<br>• Short self-efficacy for exercise scale, short outcome expectation for exercise scale, and barriers to being active quiz	NA
4	**Batalik et al. (2020)** [31]	To evaluate the use of a wrist HR monitor as a cardiac telerehabilitation device, and compare it with center-based CR	Home-based cardiac telerehabilitation:<br>• Training program: three 80-minute sessions/week consisting of 10-minute warm-up + 60-minute aerobic phase (walking or cycling, at 70–80% HRR) + 10-minute cool-down<br>• Weekly telephone calls with physiotherapist (feedback in the form of recommendations, advice, and training motivation)	12 weeks	Standard center-based CR	• VO2max<br>• Health-related quality of life (SF-36), training adherence (percentage counted from the total number of accomplished training sessions), time spent in the prescribed training HR zone, average training intensity<br>•	• **Telerehabilitation**: similar improvement of physical fitness, health-related quality of life and training adherence compared to center-based CR<br>• **VO2max** (mL/kg/min): **A) Intervention group**: from 23.7 ± 4.1 to 26.5 ± 5.7; **B) Control group**: from 23.4 ± 3.3 to 25.9 ± 4.1<br>• **Time in prescribed HR zone**: **A) Intervention group**: 53.6 ± 9.6 minutes (prescribed: 60

							minutes); average training intensity: 74.8 ± 3.4% of HRR (prescribed: 70-80% HRR); **B) Control group**: 57.1 ± 3.9 minutes (prescribed: 60 minutes); average training intensity: 75.5 ± 3.5% of HRR (prescribed: 70-80% HRR)
5	**Chung et al. (2019)** [32]	To develop and evaluate a cardiac telerehabilitation system that uses a HR monitor to support patient-provider interaction and to monitor adherence to prescribed HR zones during exercise	Home-based telerehabilitation:<br>• Prescribed HR zone: 50%–70% of HRmax; frequency/duration of the training sessions are not specified<br>• Participants wore a wearable device that provided real-time feedback on exercise intensity using LED colors (green for within prescribed target HR zone, red for above, and orange for below) and vibration alerts	3 months	No control group	• Accuracy of HR monitoring and adherence to the prescribed HR zone, as measured by the 'target heart rate retention ratio' (percentage of time within prescribed HR zone during exercise)<br>• Feasibility of the home-based telerehabilitation and the effectiveness of LED and vibration feedback to support adherence	• **Target heart rate retention ratio**: mean 92.3% ± 3.7%<br>• **Telerehabilitation:** the custom wearable device demonstrated high accuracy, with a mean absolute error of less than 2 bpm during rest, walking, and jogging; participants successfully maintained their HR within the prescribed range; real-time feedback for supporting adherence to home-based CR exercise programs was feasible
6	**Dobrican and Zampunieris (2016)** [33]	To develop and evaluate a cardiac telerehabilitation system that uses a HR monitor to monitor training intensity, adherence to target HR zones, and provide coaching	Home-based telerehabilitation:<br>• Depending on available data, this study suggests following HR training zones: (**1**) 65–90% of theoretical HRmax (220 − age) when no measured HRmax or anaerobic threshold (AT) available (this was the case for all study participants); (**2**) 55–77% theoretical HRmax (220 – age) if on medication without HRmax; (**3**) 65–90% of measured HRmax when available; (**4**) [AT−10 bpm] to [AT+5 bpm] if both HRmax and AT were known<br>• Frequency/duration of the training sessions are not specified<br>• Real-time feedback was provided when participants exceeded or fell short of their prescribed HR limits	8 weeks	No control group	• Feasibility of the HR monitoring and feedback system	• **Feasibility**: HR monitoring and feedback system functioned properly, with no data loss during the test period; the system demonstrated technical feasibility for CR<br>• No quantitative results regarding time in HR zones were presented
7	**Dosbaba et al. (2020)** [34]	To compare the effects of high-intensity interval training (HIIT) and moderate-intensity continuous training (MICT) on cardiorespiratory fitness in cardiac telerehabilitation settings	High-intensity interval training (HIIT) home-based cardiac telerehabilitation:<br>• Three 33-minute sessions/week consisting of: 5-minute warm-up at moderate intensity (ie, 65-75% of HRmax) + 4x4-minute intervals at high-intensity to reach the target HR zone (85-95% of HRmax), separated by 3-minute recoveries (at 65-75% of HRmax) + 3-minute cool-down phase<br>• App for patients (Polar Flow), dashboard for physiotherapist (Polar Flow for coach)<br>• Remote guidance/feedback via a phone call once a week	12 weeks	Moderate-intensity continuous training (MICT) home-based cardiac telerehabilitation: three 41-minute sessions/week at a continuous moderate intensity of 65-75% of HRmax, same app and feedback as HIIT group	• VO2max<br>• Health-related quality of life (SF-36), anxiety and depression (HADS), training adherence (percentage of sessions completed, time in HR zones), body composition, and safety (adverse events)	NA

8	**Quero et al. (2017)** [35]	To develop and evaluate a cardiac telerehabilitation system that uses a HR monitor for real-time PA intensity evaluation	Home-based cardiac telerehabilitation:<br>• The developed system uses a fuzzy logic algorithm fuzzy logic to classify HR into three zones (low, adequate, high intensity) based on CPET thresholds (ventilatory thresholds, HRmax, HRrest)<br>• Session duration: from 15-20 minutes building up to 30-40 minutes<br>• Provides real-time feedback via LED colors (blue/green/red), HR metrics, session progress, and a star-based adherence score; data syncs to a wearable cloud platform for remote CR monitoring.	Not disclosed, system is developed for use during home-based cardiac tele-rehabilitation but is not tested in long-term practice	No control group	• Evaluation of fuzzy logic implementation<br>• Usability of the system for home-based telerehabilitation and feedback presentation through the app	• **Feasibility**: the fuzzy logic model allowed real-time classification of HR into three intensity zones; feedback was delivered through visual (LED) and app-based mechanisms, including a star-based system summarizing zone adherence; the system was deemed suitable for home-based cardiac telerehabilitation
9	**Suchy et al. (2014)** [36] *OptimEx-CLIN*	To determine the optimal exercise dose (MICT vs. HIIT) for improving exercise capacity and diastolic function in HFpEF patients	• Supervised training program, followed by telemonitored home-based training<br>• MICT group: five 40-minute sessions/week at a continuous moderate intensity of 60–70% of HRmax<br>• HIIT group: three 38-minute sessions/week consisting of 10-minute warm-up at 50-70% of HRmax + 4x4-minute intervals at 90-95% HRmax separated by 3-minute recoveries at 50-70% of HRmax<br>• Data are sent to a telemedicine platform (OptimEx app)<br>• Non-adherence (<70% of sessions performed) triggers additional coaching and feedback from physiotherapists	12 months (3 months supervised + 9 months home-based with tele-monitoring)	Usual care	• VO2max<br>• Change in diastolic filling pressure (E/e'), and further echocardiographic and cardiopulmonary exercise testing parameters, biomarkers, quality of life, endothelial function, and adherence to training (monitored via HR and accelerometers)	NA
10	**Xia et al. (2023)** [37]	To assess the feasibility of a cardiac telerehabilitation system that uses a HR monitor to guide exercise intensity and improve adherence to prescribed training zones	Home-based cardiac telerehabilitation:<br>• Prescribed HR zone: anaerobic threshold (AT) ±5–10 bpm<br>• Frequency/duration: 30–60 minutes/day (aerobic exercise 3–5 days/week, resistance exercise 2–3 days/week at least 1 day apart, and flexibility exercise 3–5 times/week)<br>• Real-time feedback was provided when participants exceeded or fell short of their prescribed HR limits + monthly telephone calls to discuss progression and answer questions	12 weeks	No control group	• Feasibility of the home-based telerehabilitation, defined as adherence to training within prescribed HR zones for ≥10 minutes on ≥3 days per week<br>• VO2max, blood pressure, blood glucose, cholesterol, blood uric acid, left ventricle ejection fraction, quality of life (SF-12), depression and anxiety	• **Telerehabilitation:** program was feasible and resulted in significant improvements in VO2peak (+1.23 ml/kg/min), diastolic blood pressure (-4.93 mmHg), and LDL cholesterol (-0.44 mmol/L)<br>• **High adherence** (92.9%) was achieved; patients spent an average of 39.4 ± 17.8 minutes per day in the prescribed HR zone, of which 53.6% (15/28) had daily ≥30 min, 39.3% (11/28) 20–30 min, and 7.1% (2/28) less than 20 min; the average effective exercise days per week was 4.6 days (SD 2.2), of which 50.0% (14/28) were ≥5 days, 17.9% (5/28) were between 3 and 5 days, and 32.1% (9/28) were less than 3 days
11	**Koffman et al. (2023)** [38]	To identify subgroups of stroke patients based on combined HR and step data to characterize PA patterns	• Participants wore the HR monitor while continuing their normal daily routines	1 year	No control group	• Subgroup classification based on PA patterns (active, sedentary, deconditioned)<br>• Self-reported mobility, gait speed, and Stroke Impact Scale scores	• Subgroups differed significantly in PA patterns, with the deconditioned group spending more time in low steps/high HR states and showing poorer self-reported

							mobility compared to the active and sedentary groups<br>• **Time in HR zones**: most active minutes were spent in the low steps and low HR state (32±14%), followed by the high steps and high HR category (27±13%), low steps and high HR category (22±12%), and finally the high steps and low HR category (17±8%)
12	**Schubert et al. (2020)** [39]	To assess whether HR-based activity levels from wrist-worn devices can predict 6-minute walk test (6MWT) outcomes in patients with valvular heart disease	• Participants wore the HR monitor while continuing their normal daily routines<br>• Participants performed a 6MWT before the monitoring period	Average: 159 hours (~6 days with at least 23 hours monitoring per day)	No control group	• Correlation between HR-based moderate activity and 6MWT distance<br>• Prediction of achieving patient-specific 6MWT targets	• **Predicting 6MWT**: time spent in moderate activity was a significant predictor of 6MWT distance; HR-based activity levels combined with demographic factors (age, sex, BMI) explained 48% of the variance in 6MWT distances ($R^2 = 0.48$)<br>• **Median percentage of time spent in moderate activity zone**: 0.8% (IQR: 0.3–1.5%)
13	**Sandberg et al. (2016)** [40]	To compare habitual PA levels in adults with congenital heart disease with healthy age- and sex-matched controls	• Participants wore the HR monitor while continuing their normal daily routines<br>• Participants performed a submaximal step test before the monitoring period (to determine thresholds to evaluate PA)	4 days	Participants were matched by age and sex, following the same study protocol	• Comparison of PA levels between intervention and control group in different ways: 1) time spent in moderate to vigorous PA based on HR, 2) total volume of activity based on accelerometer counts per day, and 3) sedentary time periods (ie, valid HR data and no accelerometer counts)<br>• Proportion of participants meeting WHO recommendations for PA	• Congenital heart disease patients with simple lesions had higher total accelerometer counts compared to those with complex lesions and controls.<br>• **Time in moderate to vigorous PA**: similar across groups (16.0-20.3 minutes/day), with ~50% of participants meeting WHO recommendations
14	**Weeks et al. (2018)** [41]	To determine the feasibility of continuous HR monitoring in inpatient rehabilitation and assess whether patients achieved recommended HR thresholds	• Participants wore the HR monitor during their inpatient rehabilitation stay (standard-care program)	Mean: 13 days (Range: 7–28 days)	No control group	• Proportion of participants meeting HR-based PA adherence guidelines<br>• Episodes of excessive HR (>80% HRmax)<br>• Feasibility of long-term data collection (incidence of device-related skin breakdown, infections, protocol adherence), predicting characteristics of PA adherence	• **Adherence**: 60% of participants met HR-based PA adherence criteria, including the four patients with beta blocker therapy; mean duration of longest target HR bout each day: 32±22 minutes; 47% of participants experienced at least one excessive HR episode; mean longest duration of such episode: 2.4 minutes; older patients were more likely to meet PA recommendations ($R = 0.58$, $p = 0.024$)<br>• **Feasibility**: no sensor-related adverse events, no protocol deviations
15	**Brouwers et al. (2017)** [42] *SmartCare-CAD*	To evaluate if cardiac telerehabilitation using behavioral change strategies, modern communication methods and on-demand coaching will result in better long-term PA levels than center-based CR	Home-based cardiac telerehabilitation:<br>• Six supervised training sessions, followed by exercise training at home<br>• The training frequency ranges from 2–5 sessions/week, lasting 20–60 minutes at 50–80% (continuous training) or 80–90% (high intensity training) of HRR<br>• Based on principles of behavior change	12 months	Standard center-based CR	• Physical activity level (PAL, calculated from accelerometer and HR data)<br>• VO2max, BMI, blood pressure, quality of life, anxiety and depression, patient empowerment, patient satisfaction and cost effectiveness.	NA

			• App for patients, dashboard for exercise specialists (adjust goals, inspect training data, share data with other caregivers, and weekly video consultation feedback moments)				
16	**Kraal et al. (2017)** [43] *FIT@Home*	To evaluate the clinical effectiveness, cost-effectiveness, and patient satisfaction of cardiac telerehabilitation, in comparison with center-based CR	Home-based cardiac telerehabilitation:<br>• Three supervised training sessions, followed by exercise training at home<br>• The training frequency was set at at least two sessions/week, lasting 45–60 minutes at 70–85% of HRmax (continuous training)<br>• Weekly telephone coaching, based on principles of behavior change<br>• App for patients, dashboard for exercise specialists (adjust rehabilitation goals, inspect training data, share data with other caregivers)	12 weeks (+ follow-up up to 1 year after)	Standard center-based CR	• VO2max, physical activity level (PAL, calculated from accelerometer and HR data)<br>• Health related quality of life, psychosocial status, patient satisfaction, training adherence and cost-effectiveness	• **Telerehabilitation**: no differences compared to center-based CR on VO2max, physical activity level or health-related quality of life; higher patient satisfaction and more cost-effective<br>• **PAL**: **A) Intervention**: 2.09 ± 0.92 (baseline) → 2.22 ± 0.99 (discharge) → 2.14 ± 1.06 (1-year); **B) Controls**: 1.95 ± 0.86 → 2.38 ± 1.02 → 2.13 ± 1.00<br>• **Average session duration**: 64.0 ± 21.1 minutes; time within target zone (70–85% HRmax): 43.0 ± 14.8 minutes; average training intensity: 74.0 ± 3.6% of HRmax<br>• **VO2max** (mL/kg/min): **A) Intervention**: 24.4 ± 6.7 → 27.9 ± 7.5 → 27.7 ± 6.9; **B) Controls**: 24.0 ± 5.6 → 26.5 ± 7.1 → 27.5 ± 8.1
17	**Kraal et al. (2016)** [44]	To develop and validate an energy expenditure prediction model (physical activity level, PAL) for CR patients on beta-blocker medication by combining HR and accelerometer data	• Participants performed an activity protocol consisting of 11 daily activities of low to moderate intensity	A single session of an activity protocol (about 64 minutes)	No control group	• Accuracy of energy expenditure estimation (ie, physical activity level, PAL, calculated from accelerometer and HR data)<br>• Validation of the HR-flex model (personal calibration via cycling protocols) as an alternative to the multivariate regression PAL model; comparison of models with different combinations of variables (HR, accelerometer data, and patient characteristics)	• **PAL**: The highest accuracy of energy consumption estimation was achieved by the PAL model (multivariate regression model that combined HR, accelerometer data, and patient characteristics (r^2 = 0.83)).<br>• The HR-flex model, which requires individual calibration, was less accurate and less practically applicable in clinical settings
18	**Rissanen et al. (2022)** [45] *HealthBeat*	To validate a wearable HR-based method for estimating cardiorespiratory fitness during self-paced walking, comparing the estimates with CPET-measured VO2max	• Participants wore the HR monitor during a single 30-minute self-paced walk<br>• A CPET was performed to measure VO2max	30 minutes	No control group	• Agreement between estimated and measured VO2max (estimated via proprietary algorithm by Firstbeat Technologies Oy, versus measured via CPET)<br>• Subgroup analysis of accuracy in specific clinical profiles (eg, type 2 diabetes, hypertension)	• **VO2max estimates** showed a mean absolute error (MAE) of 3.1 mL/kg/min, a mean absolute percentage error (MAPE) of 10.4%, and an intraclass correlation coefficient (ICC) of 0.88 compared to CPET results<br>• The accuracy was consistent across most subgroups, but patients with type 2 diabetes had a higher error margin (MAE: 4.2 mL/kg/min; MAPE: 16.5%)
19	**Sprint et al. (2017)** [46]	To develop and apply physical activity change detection (PACD) methods to monitor HR and PA changes in inpatient rehabilitation compared to healthy individuals	• Participants wore the HR monitor during their inpatient rehabilitation stay (standard-care program)	Mean: 9.7 days (range 4–25 days)	Participants wore the HR monitor while continuing their normal daily routines for 2 weeks	• HR and PA change detection using physical activity change detection (PACD) methods in rehabilitation patients<br>• Comparison of HR-based PA patterns between rehabilitation patients and healthy individuals	• Rehabilitation patients showed significant HR and PA changes over time, particularly near discharge; healthy control group showed stable PA; HR-based PA assessment provided valuable insights beyond step count alone

							• **Average time in the target HR zone** = 9.7 ± 5.8 minutes/day
**20**	**Hannan et al. (2021)** [47]	To determine whether monitoring PAI (personal activity intelligence) influences the amount and/or intensity of PA in the maintenance phase of CR and to explore participants' perceptions of this approach	• Participants wore the HR monitor in their daily life: first 3 weeks blinded + afterwards participants had access to their PAI-scores via an application and received education on the PAI system<br>• Participants could choose the location, time, and amount of exercise	6 weeks	No separate control group; participants served as their own controls (blinded phase vs unblinded phase)	• Change in PAI score after participants were unblinded and could see their data<br>• Quality of life (EQ-5D-5L score)	• **PAI**: 89% of participants improved their total PAI score after receiving access to their data; the number of participants achieving ≥50 PAI per week increased from 39% to 61%<br>• Participants found PAI monitoring motivating and appreciated the feedback; quality of life improved, with EQ-5D-5L scores increasing by 0.6 ± 1.05 (p = 0.019)

CR: cardiac rehabilitation; PA: physical activity; HR: heart rate; HRR: heart rate reserve, calculated as HRR = HRmax – HRrest; PAL: physical activity level; VO2max: maximal oxygen update; EQ-5D: euroQol 5-dimension questionnaire; HADS: hospital anxiety and depression scale; SF-36: 36-item short form health survey; SF-12: 12-item short form health survey; BMI: body mass index; HIIT: high-intensity interval training; MICT: moderate-intensity continuous training; HFpEF: heart failure with preserved ejection fraction; HFrEF: heart failure with reduced ejection fraction; CPET: cardiopulmonary exercise testing; 6MWT: 6-minute walk test; PACD: physical activity change detection; PAI: personal activity intelligence: AT: anaerobic threshold.

References:

28. *Alonso, W.W., et al., The HEART Camp Exercise Intervention Improves Exercise Adherence, Physical Function, and Patient-Reported Outcomes in Adults With Preserved Ejection Fraction Heart Failure. Journal of cardiac failure, 2021. 28(3): p. 431.*

29. *Crozier, A., et al., Mobile Health for Cardiac Rehabilitation (Motivate-CR+). Mobile Health Biometrics to Prescribe Immediate Remote Physical Activity for Enhancing Uptake to Cardiac Rehabilitation, 2023. 109: p. A14-A15.*

30. *Mansfield, A., et al., Promoting Optimal Physical Exercise for Life (PROPEL): aerobic exercise and self-management early after stroke to increase daily physical activity-study protocol for a stepped-wedge randomised trial. BMJ Open, 2017. 7(6): p. e015843.*

31. *Batalik, L., et al., Benefits and effectiveness of using a wrist heart rate monitor as a telerehabilitation device in cardiac patients: a randomized controlled trial. Medicine, 2020. 99(11): p. e19556.*

32. *Chung, H., et al., Patient-Provider Interaction System for Efficient Home-Based Cardiac Rehabilitation Exercise. IEEE ACCESS, 2019. 7: p. 14611-14622.*

33. *Dobrican, R.A., D. Zampunieris, and Ieee, A Proactive Solution, using Wearable and Mobile Applications, for Closing the Gap between the Rehabilitation Team and Cardiac Patients. 2016. p. 146-155.*

34. *Dosbaba, F., et al., Effect of home-based high-intensity interval training using telerehabilitation among coronary heart disease patients. Medicine, 2020. 99(47): p. e23126.*

35. *Quero, J.M., et al., Real-Time Monitoring in Home-Based Cardiac Rehabilitation Using Wrist-Worn Heart Rate Devices. SENSORS, 2017. 17(12).*

36. *Suchy, C., et al., Optimising exercise training in prevention and treatment of diastolic heart failure (OptimEx-CLIN): rationale and design of a prospective, randomised, controlled trial. European journal of preventive cardiology, 2014. 21(2): p. 18.*

37. *Xia, K., et al., Feasibility of a smartphone app for prescribed exercise tutoring in patients with stable coronary heart disease. Digit Health, 2023. 9: p. 20552076231197424.*

38. *Koffman, L.J., et al., Identifying Unique Subgroups of Individuals With Stroke Using Heart Rate and Steps to Characterize Physical Activity. J Am Heart Assoc, 2023. 12(18): p. e030577.*

39. *Schubert, C., et al., Wearable devices can predict the outcome of standardized 6-minute walk tests in heart disease. NPJ Digit Med, 2020. 3: p. 92.*

40. *Sandberg, C., et al., Habitual Physical Activity in Adults With Congenital Heart Disease Compared With Age- and Sex-Matched Controls. Can J Cardiol, 2016. 32(4): p. 547-53.*

41. *Weeks, D.L., et al., Implementing Wearable Sensors for Continuous Assessment of Daytime Heart Rate Response in Inpatient Rehabilitation. Telemed J E Health, 2018. 24(12): p. 1014-20.*
42. *Brouwers, R.W., et al., Effects of cardiac telerehabilitation in patients with coronary artery disease using a personalised patient-centred web application: protocol for the SmartCare-CAD randomised controlled trial. BMC cardiovascular disorders, 2017. 17(1): p. 46.*
43. *Kraal, J.J., et al., Clinical and cost-effectiveness of home-based cardiac rehabilitation compared to conventional, centre-based cardiac rehabilitation: Results of the FIT@Home study. European Journal of Preventive Cardiology, 2017. 24(12): p. 1260-1273.*
44. *Kraal, J.J., et al., Energy expenditure estimation in beta-blocker-medicated cardiac patients by combining heart rate and body movement data. Eur J Prev Cardiol, 2016. 23(16): p. 1734-1742.*
45. *Rissanen, A.E., et al., Cardiorespiratory Fitness Estimation Based on Heart Rate and Body Acceleration in Adults With Cardiovascular Risk Factors: Validation Study. JMIR Cardio, 2022. 6(2): p. e35796.*
46. *Sprint, G., et al., Analyzing Sensor-Based Time Series Data to Track Changes in Physical Activity during Inpatient Rehabilitation. Sensors (Basel), 2017. 17(10).*
47. *Hannan, A.L., et al., Effect of personal activity intelligence (PAI) monitoring in the maintenance phase of cardiac rehabilitation: a mixed methods evaluation. BMC Sports Sci Med Rehabil, 2021. 13(1): p. 124.*

Table S3. Overview of retrospective cohort studies estimating PAI scores from self-reported physical activity questionnaires (without heart rate monitoring).

ID	Title	First author (year, study country)	Name population study (data collection period)	Sample size	Population	Gender distribution	Age (years,mean ± SD/median)[a]	BMI (kg/m², mean±SD/median)[a]	Aim study	Outcome	Main findings
a	Personalized Activity Intelligence (PAI) for prevention of cardiovascular disease and promotion of physical activity	Nes (2016, Norway)	HUNT3 (2006-2008)	39,298	General population	20,042 female (51.0%), 19.256 male (49.0%)	42.6 ± 14.1	Mean not explicitly reported, but if BMI ≥25: 28.0 ± 3.0	To derive and validate a single metric of activity tracking (ie, PAI) that associates with lower risk of cardiovascular disease mortality	CVD mortality, all-cause mortality	PAI ≥100 was associated with a 17% (95% CI: 7–27%) reduction in CVD mortality for men and 23% (95% CI: 4–38%) for women. The corresponding all-cause mortality reductions were 13% and 17%, respectively.
b	Personal Activity Intelligence (PAI), sedentary behavior and cardiovascular risk factor clustering – the HUNT Study	Zisko (2017, Norway)	HUNT3 (2006-2008)	29,950	General population (individuals with a history of CVD, stroke, diabetes mellitus, or antihypertensiva use were excluded)	16,054 female (53.6%), 13,896 male (46.4%)	Varied between 45.7 ± 13.6 and 51.4 ± 14.7 depending on the PAI classification	Mean varied between 26.1 ± 3.8 and 27.2 ± 5.0 depending on PAI classification	To investigate whether PAI (≥100) mitigates the negative effect of sedentary behavior on the clustering of cardiovascular risk factors	Association between PAI, sedentary behavior, and cardiovascular risk factor clustering	PAI ≥100 was associated with lower odds of CV risk factor clustering (OR for PAI=0: 1.76, 95% CI: 1.40–2.20). Higher PAI attenuated the impact of sedentary behavior, particularly in those aged ≥60 years (OR: 2.29, 95% CI: 1.53–3.44 if PAI <100).
c	Personal Activity Intelligence and mortality in patients with cardiovascular disease: the HUNT study	Kieffer (2018, Norway)	HUNT1 (1984-1986, follow-up until 2015)	3,133	Participants with self-reported angina pectoris, myocardial infarction, or stroke	1,128 female (36.0%), 2,005 male (64.0%)	67.6 ± 10.3	Mean not explicitly reported, but BMI ≥30 in 11.3%-19.2% of subgroups	To investigate whether PAI (≥100) is associated with lower CVD and all-cause mortality in patients with CVD, and whether these associations depend on adherence to PA recommendations	CVD mortality, all-cause mortality	PAI ≥100 reduced CVD mortality by 36% (HR: 0.64, 95% CI: 0.52–0.79) and all-cause mortality by 24% (HR: 0.76, 95% CI: 0.65–0.90). Benefits were observed across sex, age, and BMI groups. Meeting PA adherence guidelines was not necessary to achieve these benefits.
d	Temporal changes in a novel metric of physical activity tracking (Personal Activity Intelligence) and mortality: the HUNT study, Norway	Kieffer (2019, Norway)	HUNT1 (1984-1986), HUNT2 (1995-1997), follow-up until 2015	24,880	General population (individuals with self-reported CVD were excluded)	13,010 female (52.3%), 11,870 male (47.7%)	46.9 ± 12.8	Mean not explicitly reported, but BMI ≥30 in 10.6%-12.1% of subgroups	To investigate whether temporal changes in PAI over 10 years are associated with CVD and all-cause mortality	CVD mortality, all-cause mortality	Maintaining PAI ≥100 was associated with 32% lower CVD mortality (AHR: 0.68, 95% CI: 0.54–0.86) and 20% lower all-cause mortality (AHR: 0.80, 95% CI: 0.71–0.91). Increasing PAI from <100 to ≥100 was linked to 6.6 additional years of life.
e	Personal Activity Intelligence (PAI): a new standard in activity tracking for obtaining a healthy cardiorespiratory fitness level and low cardiovascular risk	Nauman (2019, Norway)	HUNT3 (2006-2008)	4,334	General population (individuals with a history of CVD, stroke, diabetes mellitus, or antihypertensiva use were excluded)	2,222 female (51.3%), 2,112 male (48.7%)	48.5 ±13.5	25.9 ± 3.6	To summarize epidemiological evidence on PAI and its effects on health outcomes, and to explore the association between PAI and VO2max	Association between PAI and VO2max	Men with PAI ≥100 had VO2peak values 5.4 mL·kg⁻¹·min⁻¹ higher (95% CI: 4.5–6.3), and women 4.1 mL·kg⁻¹·min⁻¹ higher (95% CI: 3.1–5.2) compared to inactive peers.
f	Temporal changes in personal activity intelligence and mortality:	Nauman (2021, United	Aerobics Center Longitudinal	17,613	General population (individuals with self-	3,242 female (18.4%),	Varied between 48.5 ± 9.4 and 53.1	Mean not explicitly reported, but BMI	To investigate whether temporal changes in PAI over time are associated with CVD	CVD mortality, all-cause mortality	Participants maintaining PAI ≥100 had 51% lower CVD mortality (aHR: 0.49, CI: 0.26–0.95) and 42% lower

	data from the aerobics center longitudinal study	States of America)	study (1974–2002, follow-up until 2003)		reported CVD were excluded)	14,371 male (81.6%)	± 10.6 depending on PAI classification	≥30 in 7.2%-19.8% of subgroups	and all-cause mortality in a large United States population		all-cause mortality (aHR: 0.58, CI: 0.41–0.83). Life expectancy gains were 4.8 years for sustained ≥100 PAI.
**g**	Personal Activity Intelligence and ischemic heart disease in a healthy population: China Kadoorie Biobank Study	Hammer (2022, China)	China Kadoorie Biobank study (2004–2008, follow-up until 2015)	443,792	General population (individuals with self-reported CVD were excluded)	266,263 female (60.0%), 177,529 male (40.0%)	50.9 ± 10.2	24.0 ± 3.4	To determine whether PAI-score is a predictor of fatal ischemic heart disease and nonfatal myocardial infarction in a large Chinese cohort	Ischemic heart disease mortality, nonfatal myocardial infarction	PAI ≥100 was associated with a 9% reduction in fatal ischemic heart disease (aHR: 0.91, CI: 0.83–1.00) and 6% in nonfatal myocardial infarction (aHR: 0.94, CI: 0.83–1.05). Stronger effects were observed in those aged ≥60 (ischemic heart disease: 16% reduction, myocardial infarction: 13% reduction).
**h**	Temporal changes in Personal Activity Intelligence and the risk of incident dementia and dementia related mortality: a prospective cohort study (HUNT)	Tari (2022, Norway)	HUNT1 (1984-1986), HUNT2 (1995-1997), follow-up until 2021	29,826	General population (individuals with self-reported history of myocardial infarction or stroke were excluded)	15,577 female (52.2%); 14,249 male (47.8%)	Varied between 49.6 ± 11.5 and 55.1 ± 13.4 depending on the HUNT number and PAI classification	Varied between 25.7 ± 3.1 and 26.8 ± 4.1 depending on HUNT number and PAI classification	To investigate whether changes in PAI scores over time are associated with the risk of incident dementia and dementia-related mortality in a large Norwegian population	Incident dementia, dementia-related mortality, years of life gained dementia-free	Maintaining PAI ≥100 reduced dementia risk by 25% (aHR: 0.75, 95% CI: 0.58–0.97) and dementia-related death by 38% (aHR: 0.62, 95% CI: 0.43–0.91). Gains included ~2.8 dementia-free years and ~2.4 additional life years.

All studies listed in this table were excluded from the main review because PAI was estimated retrospectively using self-reported PA questionnaires rather than based on HR monitoring. Participants were followed over time to evaluate associations between estimated PAI levels and health outcomes such as cardiovascular disease, mortality, or cardiorespiratory fitness. Beta-blocker use was not reported in any of the included studies.

PAI: Personal Activity Intelligence; CVD: cardiovascular disease; VO2max: maximal oxygen uptake; SD: standard deviation; CI: confidence interval; HR: hazard ratio; OR: odds ratio; aHR: adjusted hazard ratio.

a: for each study, age/BMI was reported as originally provided by the authors. If age/BMI was available for the total study population, this value was reported. If only group-specific data were available, separate values are shown.
