# Supplementary material for: Heart Rate Monitors for the Estimation of Physical Activity in Patients With Cardiovascular Disease: Systematic Review
Source: JMIR Mhealth Uhealth. 2026 Jun 17;14:e79995. doi: 10.2196/79995 (PMC13274969; doi:10.2196/79995)
Supplement: Checklist 1 [file mhealth-v14-e79995-s002.pdf]

PRISMA 2020 Checklist

Section and Topic	Item #	Checklist item	Location where item is reported
**TITLE**			
Title	1	Identify the report as a systematic review.	"Heart Rate Monitors for the Estimation of Physical Activity in Patients with Cardiovascular Disease: A Systematic Review"
**ABSTRACT**			
Abstract	2	See the PRISMA 2020 for Abstracts checklist.	The abstract provides a structured summary in accordance with the PRISMA 2020 for Abstracts checklist. It includes the review's objectives, eligibility criteria, information sources, methods for risk of bias assessment and narrative synthesis, and the number of included studies. Main findings are summarized across four identified HR-based estimation approaches, followed by a conclusion highlighting the clinical potential of HR-based activity monitoring in cardiovascular care. Full details are presented in the Abstract section of the manuscript.
**INTRODUCTION**			
Rationale	3	Describe the rationale for the review in the context of existing knowledge.	The rationale for the review is provided in the Introduction section of the manuscript. It describes the current state of knowledge regarding physical activity (PA) monitoring in patients with cardiovascular disease (CVD), highlights limitations of traditional and commercially available methods, and explains the potential advantages of heart rate (HR)–based monitoring. The Introduction also notes the lack of previous systematic reviews summarizing HR-based PA estimation methods in cardiac populations, underscoring the need for this review.
Objectives	4	Provide an explicit statement of the objective(s) or question(s) the review addresses.	The main objective of the review is to describe how wearable heart rate monitors have been used to quantify physical activity in adult patients with cardiovascular disease. The objective is clearly stated in the Abstract and elaborated in the final paragraph of the Introduction section.
**METHODS**			
Eligibility criteria	5	Specify the inclusion and exclusion criteria for the review and how studies were grouped for the syntheses.	The eligibility criteria are described in the Methods section 2.2 (Eligibility Criteria), using a PICO-informed approach. Population: Adults (≥18 years) with cardiovascular disease (CVD) or related cardiovascular risk factors. Intervention: Use of wearable heart rate monitors (e.g., wrist-worn PPG or chest-strap ECG) to estimate or quantify physical activity. Comparator/Outcome: No specific comparator was required. Studies had to report methods for deriving physical activity metrics (e.g., time in HR zones, energy expenditure, VO2max estimation) from HR data. Additional eligibility features included: original research or study protocols, publication between 2014 and 2024, English language, and full-text availability. Studies were excluded if HR data were only used for exercise prescription (e.g., training zone targets) without quantifying PA, if they did not target (or explicitly refer to) cardiac populations, or if they were reviews, editorials, or conference abstracts.
Information sources	6	Specify all databases, registers, websites, organisations, reference lists and other sources searched or consulted to identify studies. Specify the date when each source was last searched or consulted.	All information sources used to identify studies are described in Section 2.1 (Search Strategy). A comprehensive search was conducted in the following bibliographic databases: PubMed, Web of Science, and the Cochrane Central Register of Controlled Trials (CENTRAL). The final search was executed on April 3, 2024. Additionally, backward citation searches were performed by screening the reference lists of included articles.
Search strategy	7	Present the full search strategies for all databases, registers and websites, including any filters and limits used.	Full search strategies for each database are provided in Multimedia Appendix 1, Table S1.

PRISMA 2020 Checklist

Section and Topic	Item #	Checklist item	Location where item is reported
Selection process	8	Specify the methods used to decide whether a study met the inclusion criteria of the review, including how many reviewers screened each record and each report retrieved, whether they worked independently, and if applicable, details of automation tools used in the process.	The screening process is described in Section 2.3 (Screening and Data Management). Two reviewers independently screened all records at the title, abstract, and full-text stages using Rayyan software. Discrepancies were resolved through discussion, or by consulting a third reviewer when necessary. No automation tools were used, and no translation was required during study selection.
Data collection process	9	Specify the methods used to collect data from reports, including how many reviewers collected data from each report, whether they worked independently, any processes for obtaining or confirming data from study investigators, and if applicable, details of automation tools used in the process.	The methods used to collect data are described in Section 2.4 (Data Extraction). Two reviewers independently extracted data using a structured Excel form, which was pilot-tested on two studies for consistency and clarity. Discrepancies were resolved through consensus discussions or, if needed, by consulting a third reviewer. No automation tools were used, and no translation was required during data collection.
Data items	10a	List and define all outcomes for which data were sought. Specify whether all results that were compatible with each outcome domain in each study were sought (e.g. for all measures, time points, analyses), and if not, the methods used to decide which results to collect.	All outcomes for which data were sought are defined in Section 2.4 (Data Extraction). The primary outcome of interest was the methodological approach used to estimate physical activity (PA) using heart rate data (detailed in Table 2).
	10b	List and define all other variables for which data were sought (e.g. participant and intervention characteristics, funding sources). Describe any assumptions made about any missing or unclear information.	As described in Section 2.4 (Data Extraction), the following additional variables were collected: study characteristics (authors, year, design, country, funding), participant demographics (age, sex, BMI, diagnosis group, beta-blocker use), HR monitor specifications (brand, device type, measurement method), details of the intervention or monitoring context (e.g. duration, training program, feedback mechanisms), and a summary of the main findings. No imputation or assumptions were made for missing or unclear data; discrepancies were discussed between reviewers and resolved by consensus.
Study risk of bias assessment	11	Specify the methods used to assess risk of bias in the included studies, including details of the tool(s) used, how many reviewers assessed each study and whether they worked independently, and if applicable, details of automation tools used in the process.	The methods used to assess risk of bias are described in Section 2.5 (Quality and Risk of Bias Assessment). The Crowe Critical Appraisal Tool (CCAT) was used to evaluate methodological quality across eight domains. Two reviewers independently assessed each study and resolved any discrepancies through consensus. For protocol papers, only applicable domains were scored, and total scores were recalculated as a percentage of the relevant maximum. No automation tools were used in this process.
Effect measures	12	Specify for each outcome the effect measure(s) (e.g. risk ratio, mean difference) used in the synthesis or presentation of results.	No formal effect measures were defined, as this review used a narrative synthesis approach. The focus was on describing methodological approaches to heart rate–based physical activity estimation. Where individual studies reported validation statistics (e.g., mean absolute error), these were presented descriptively but were not pooled or synthesized across studies.
Synthesis methods	13a	Describe the processes used to decide which studies were eligible for each synthesis (e.g. tabulating the study intervention characteristics and comparing against the planned groups for each synthesis (item #5)).	As described in Section 2.6 (Data Synthesis), all included studies were eligible for narrative synthesis. Studies were grouped post hoc based on their methodological approach to heart rate–based physical activity estimation. This grouping resulted in four categories: (1) HR zone analysis, (2) physiological modelling, (3) PA change detection, and (4) personalized scoring systems. This classification guided the structure of the results synthesis, as elaborated in Section 3.5 and illustrated in Figure 2.
	13b	Describe any methods required to prepare the data for presentation or synthesis, such as handling of missing summary statistics, or data	No statistical transformations or data conversions were required, as the review used a narrative synthesis approach (Section 2.6, Data Synthesis). As part of the

Section and Topic	Item #	Checklist item	Location where item is reported
		conversions.	data preparation, studies were classified post hoc into four methodological categories based on how heart rate data were used to estimate physical activity. Missing or unreported data were noted as such, and no imputation or standardization was performed.
	13c	Describe any methods used to tabulate or visually display results of individual studies and syntheses.	Although no formal statistical synthesis was conducted, the review employed structured tabulation and visual grouping to support narrative synthesis. As part of the synthesis planning (Section 2.6, Data Synthesis), studies were organized into four methodological categories, which guided the layout of Tables and Figures. Results were displayed in Table 1 (study and population characteristics), Table 2 (HR-based PA estimation approach), and Table 3 (intervention/observation context). Figure 2 provides a graphical summary of the methodological classification across studies.
	13d	Describe any methods used to synthesize results and provide a rationale for the choice(s). If meta-analysis was performed, describe the model(s), method(s) to identify the presence and extent of statistical heterogeneity, and software package(s) used.	As stated in Section 2.6 (Data Synthesis), a narrative synthesis approach was used due to heterogeneity in study designs, populations, HR monitor types, and outcome definitions. No statistical synthesis or meta-analysis was performed. Therefore, no models, effect estimates, or statistical heterogeneity measures were applied.
	13e	Describe any methods used to explore possible causes of heterogeneity among study results (e.g. subgroup analysis, meta-regression).	No statistical methods (eg, subgroup analysis or meta-regression) were used to explore heterogeneity, as no effect estimates were synthesized (Section 2.6, Data Synthesis). However, methodological heterogeneity was explored qualitatively through post hoc classification of included studies into four distinct HR-based PA estimation approaches. Differences between these approaches (eg, zone definitions, model inputs, validation procedures, feedback mechanisms) are discussed in Sections 3.5 and 4.1–4.4.
	13f	Describe any sensitivity analyses conducted to assess robustness of the synthesized results.	No sensitivity analyses were conducted, as the review used a narrative synthesis approach without pooling of effect estimates (Section 2.6, Data Synthesis). The methodological classification was determined retrospectively and applied consistently across all included studies.
Reporting bias assessment	14	Describe any methods used to assess risk of bias due to missing results in a synthesis (arising from reporting biases).	No formal assessment of risk of bias due to missing results (eg, publication bias or selective outcome reporting) was conducted, as no statistical synthesis or meta-analysis was performed. Reporting limitations of individual studies were reflected in the CCAT-based quality appraisal (Section 2.5).
Certainty assessment	15	Describe any methods used to assess certainty (or confidence) in the body of evidence for an outcome.	No formal assessment of certainty in the body of evidence (eg, using GRADE) was performed. As this review focused on mapping methodological approaches rather than synthesizing intervention effects or outcome estimates, a certainty framework was not applicable. Methodological quality was assessed at the individual study level using the Crowe Critical Appraisal Tool, as described in Section 2.5.
**RESULTS**			
Study selection	16a	Describe the results of the search and selection process, from the number of records identified in the search to the number of studies included in the review, ideally using a flow diagram.	The search and selection process is reported in Section 3.1 (Study Selection Process) and illustrated in Figure 1 (PRISMA flow diagram). Of 828 records identified, 673 remained after de-duplication. After title and abstract screening, 53 full texts were reviewed, resulting in 20 studies included in the review.
	16b	Cite studies that might appear to meet the inclusion criteria, but which were excluded, and explain why they were excluded.	Studies that appeared to meet inclusion criteria but were excluded are discussed in Section 4.4 (Personal Activity Intelligence (PAI): Validation, Challenges, and Future Directions). Eight studies focusing on the PAI scoring system were excluded

Section and Topic	Item #	Checklist item	Location where item is reported
			because they did not use data from wearable heart rate monitors. These studies initially seemed highly relevant, as they examined a heart rate–based PA scoring method in relation to cardiovascular outcomes. However, upon closer inspection, PAI was retrospectively estimated from self-reported physical activity questionnaires rather than actual HR data. Although the PAI system was designed for use with HR monitors, this was not yet implemented in these retrospective validation studies. The excluded studies and reasons are listed in Table S2 in Multimedia Appendix 1.
Study characteristics	17	Cite each included study and present its characteristics.	All included studies are cited and presented in the Results section and in detail in Tables 1–3. Table 1 presents general study characteristics and participant demographics (eg, design, sample size, age, sex, diagnosis, medication use). Table 2 summarizes the heart rate–based physical activity estimation methods per study. Table 3 provides information on the intervention or observational context (eg, program duration, feedback mechanisms, study aims). Together, these tables enable comparison of key characteristics across studies.
Risk of bias in studies	18	Present assessments of risk of bias for each included study.	The quality and risk of bias of each included study were assessed using the Crowe Critical Appraisal Tool (CCAT), as described in Section 2.5. Results of the assessment are presented in Section 3.6. Each study was scored across eight domains, with total scores ranging from 50% to 87%. A summary of domain-specific performance is provided (eg, strong scores in data collection and discussion, weaker scores in sampling and ethics). CCAT scores are summarized in Table 4.
Results of individual studies	19	For all outcomes, present, for each study: (a) summary statistics for each group (where appropriate) and (b) an effect estimate and its precision (e.g. confidence/credible interval), ideally using structured tables or plots.	Individual study findings are presented in structured format in Tables 2 and 3, and described throughout Section 3.5. As no effect sizes were synthesized, no summary statistics or confidence intervals are reported across studies. Where available, study-specific metrics (eg, time in HR zones, adherence percentages, VO2max estimation accuracy) were extracted and presented descriptively. All data reflect the original reporting of each study.
Results of syntheses	20a	For each synthesis, briefly summarise the characteristics and risk of bias among contributing studies.	Study characteristics and methodological quality within each synthesis group are summarized in Sections 3.5 and 3.6. Studies were grouped into four methodological categories based on their approach to HR-based PA estimation. Risk of bias was assessed using the Crowe Critical Appraisal Tool (Section 2.5), and results are presented in Section 3.6. Most studies showed moderate to high methodological quality, though limitations in sampling, ethics reporting, and external validation were noted.
	20b	Present results of all statistical syntheses conducted. If meta-analysis was done, present for each the summary estimate and its precision (e.g. confidence/credible interval) and measures of statistical heterogeneity. If comparing groups, describe the direction of the effect.	No statistical syntheses or meta-analyses were conducted. As the review focused on mapping methodological approaches to HR-based physical activity estimation, a narrative synthesis was performed (Section 2.6, Data Synthesis). Individual study findings are presented descriptively in Sections 3.5 and 3.6.
	20c	Present results of all investigations of possible causes of heterogeneity among study results.	No formal statistical analyses were performed to explore heterogeneity (eg, subgroup analysis or meta-regression). However, methodological heterogeneity was examined qualitatively across studies in Sections 3.5 and 4.1–4.4. Variations in heart rate zone definitions, device types, modeling inputs, validation strategies, and feedback mechanisms were identified and discussed.

PRISMA 2020 Checklist

Section and Topic	Item #	Checklist item	Location where item is reported
	20d	Present results of all sensitivity analyses conducted to assess the robustness of the synthesized results.	No sensitivity analyses were conducted. As no statistical synthesis or pooled effect estimates were generated, sensitivity testing was not applicable.
Reporting biases	21	Present assessments of risk of bias due to missing results (arising from reporting biases) for each synthesis assessed.	No formal assessment of risk of bias due to missing results was conducted. As the review did not include statistical syntheses or meta-analyses, evaluation of reporting bias (eg, through funnel plots or outcome reporting comparisons) was not applicable. Limitations in reporting at the individual study level were captured through CCAT-based quality appraisal (see Sections 2.5 and 3.6).
Certainty of evidence	22	Present assessments of certainty (or confidence) in the body of evidence for each outcome assessed.	No formal assessment of certainty in the body of evidence was conducted (eg, using GRADE). As the review focused on mapping methodological approaches rather than synthesizing intervention effects or outcome data, a certainty framework was not applicable. Methodological quality of individual studies was assessed using the CCAT (Sections 2.5 and 3.6), and the limitations of the evidence are discussed in the Discussion.
**DISCUSSION**			
Discussion	23a	Provide a general interpretation of the results in the context of other evidence.	A general interpretation of the results is provided in Section 4 (Discussion), including Subsections 4.1–4.4. The findings are discussed in the context of existing evidence on physical activity monitoring and heart rate–based estimation approaches. The review highlights methodological strengths and gaps, compares HR monitoring with conventional tools such as accelerometry and questionnaires, and evaluates emerging systems like PAI and PACD in relation to their clinical utility and evidence base.
	23b	Discuss any limitations of the evidence included in the review.	The limitations of the included evidence are discussed throughout the Discussion. Key limitations include lack of external validation for several estimation methods, variability in HR zone definitions and calculation methods, and limited algorithmic transparency. Some approaches, such as PAI and VO2max modeling, rely on proprietary algorithms that hinder reproducibility. Some methods have not yet been validated in older adults or patients with complex cardiovascular conditions.
	23c	Discuss any limitations of the review processes used.	The limitations of the review process are addressed in Section 4.5 (Future Directions and Research Limitations). Due to the methodological diversity of included studies, no meta-analysis was performed. Although the search was comprehensive, it was limited to publications available by April 3, 2024.
	23d	Discuss implications of the results for practice, policy, and future research.	mplications for practice and research are discussed in detail in Section 4.5 (Future Directions and Research Limitations) and summarized in Section 5 (Conclusions). The review highlights the clinical potential of heart rate–based physical activity estimation in cardiovascular care, especially in supporting personalized guidance beyond traditional step counts or questionnaires. However, methodological diversity, lack of validation, and algorithmic opacity currently limit implementation. Future research should prioritize external validation in clinical populations, algorithm transparency, and development of scalable, feedback-oriented tools suitable for long-term monitoring and clinical integration.
**OTHER INFORMATION**			
Registration and protocol	24a	Provide registration information for the review, including register name and registration number, or state that the review was not registered.	This review was not registered in a systematic review registry. This was stated in the beginning of the Methods section.

PRISMA 2020 Checklist

Section and Topic	Item #	Checklist item	Location where item is reported
	24b	Indicate where the review protocol can be accessed, or state that a protocol was not prepared.	A review protocol was developed by the authors to guide the review process, but it was not publicly registered or published. This was stated in the beginning of the Methods section.
	24c	Describe and explain any amendments to information provided at registration or in the protocol.	No amendments to a registered protocol or public record were made.
Support	25	Describe sources of financial or non-financial support for the review, and the role of the funders or sponsors in the review.	No financial or non-financial support was received for conducting this review. This is stated under the "Funding" section of the manuscript.
Competing interests	26	Declare any competing interests of review authors.	The author(s) declared no potential conflicts of interest with respect to the research, authorship, and/or publication of this article. This is stated under the “Conflicts of Interest” section of the manuscript.
Availability of data, code and other materials	27	Report which of the following are publicly available and where they can be found: template data collection forms; data extracted from included studies; data used for all analyses; analytic code; any other materials used in the review.	A data availability statement is included in the manuscript. It states: “Raw data supporting the conclusions of this article will be made available by the authors upon request.”

From: Page MJ, McKenzie JE, Bossuyt PM, Boutron I, Hoffmann TC, Mulrow CD, et al. The PRISMA 2020 statement: an updated guideline for reporting systematic reviews. BMJ 2021;372:n71. doi: 10.1136/bmj.n71.
This work is licensed under CC BY 4.0. To view a copy of this license, visit https://creativecommons.org/licenses/by/4.0/
